# Estrogen attenuates thoracic aortic aneurysm progression through reduced extracellular matrix remodeling and wall stiffening in a severe Marfan syndrome mouse model

**DOI:** 10.3389/fphys.2026.1944714

**Published:** 2026-10-30

**Authors:** Krashn Kumar Dwivedi, Yufan Wu, Jessica E. Wagenseil

**Affiliations:** 1 Department of Mechanical Engineering and Materials Science, Washington University, Saint Louis, MO, United States; 2 Department of Biomedical Engineering, University of Utah, Salt Lake, UT, United States

**Keywords:** aneurysm, aortic stiffness, estrogen (17β-estradiol), extracellular matrix, Marfan syndrome

## Abstract

Thoracic aortic aneurysm (TAA) is a life-threatening cardiovascular disease characterized by progressive aortic dilation and fatal dissection or rupture. Marfan syndrome (MFS)-associated TAA exhibits sexual dimorphism, suggesting a role for sex hormones. Although 17β-estradiol attenuates aortic root dilation in male MFS mice, the contribution of endogenous estrogen to sex-specific TAA progression and its effects on aortic wall extracellular matrix (ECM) remodeling and wall stiffness remains poorly understood. Using a severe MFS mouse model (*Fbn1^mgR/mgR^*) with TAA formation and reduced lifespan, male mice received 17β-estradiol or placebo pellets and female mice underwent bilateral ovariectomy (OVX) or sham surgery, starting at one month of age. *In vivo* ascending thoracic aortic (ATA) geometry and wall stiffness were assessed by ultrasound imaging and ECM remodeling and α-SMA protein amount were evaluated by multiphoton imaging and immunofluorescence, respectively. 17β-estradiol-treated male and sham female mice exhibited slow ATA dilation, preserved wall stiffness, and prolonged survival, whereas placebo-treated male and OVX female mice showed fast ATA dilation, age-dependent wall stiffening, and reduced survival. Histological analysis demonstrated marked elastic fiber fragmentation in the ECM and loss of α-SMA amounts in placebo-treated male and OVX female mice, while 17β-estradiol-treated male and sham female mice displayed intact elastic fibers and preserved α-SMA expression. Overall, these findings demonstrate a protective role of estrogen in maintaining ECM integrity, aortic wall stiffness, and vascular smooth muscle cell phenotype, thereby attenuating ATA progression and improving long-term survival in MFS mice.

## 1 Introduction

Thoracic aortic aneurysm (TAA) is a serious cardiovascular disorder that affects approximately 5.3 individuals per 100,000 population annually (Gouveia e Melo et al., 2022). TAA is characterized by progressive enlargement of the thoracic aorta, resulting in abnormal dilation of the vessel wall (Gouveia e Melo et al., 2022). As the disease advances, the aortic wall becomes increasingly vulnerable to aortic dissection and rupture, both of which are associated with high morbidity and mortality if left untreated (Gouveia e Melo et al., 2022). TAA is associated with genetic disorders such as Marfan syndrome (MFS) (Milewicz et al., 2008), a systemic genetic disease caused by mutations in the fibrillin-1 (FBN1) gene (Pereira et al., 1997). Fibrillin-1 is an extracellular matrix (ECM) glycoprotein that plays a critical role in the assembly, organization, and long-term structural integrity of elastic fibers within the aortic wall (Ramirez and Dietz, 2007). Mutations in FBN1 disrupt normal elastic fiber formation and maintenance, leading to extensive elastic fiber fragmentation and degeneration (Ramirez and Dietz, 2007). In addition, FBN1 deficiency contributes to vascular smooth muscle cell (vSMC) dysfunction, aberrant activation of transforming growth factor-beta (TGF-β) signaling pathways, and progressive medial fibrosis (Seeburun et al., 2023; Dwivedi et al., 2025). Collectively, these pathological alterations compromise the structural and mechanical integrity of the aortic wall, leading to aortic dilation and dissection/rupture (Bollache et al., 2018).

Epidemiological studies indicate that despite equal prevalence of MFS in men and women, men remain at higher risk of TAA development, progression, and aortic events (dissection or rupture) at younger ages, whereas women show protective responses toward TAA growth and its adverse events (Detaint et al., 2010; Roman et al., 2017). In women, this protection diminishes in older age when they experience an increased risk of TAA progression and aortic events (Nienaber et al., 2004; Detaint et al., 2010). The marked sex differences observed in TAA development, progression, and clinical outcomes suggest an important role for sex hormones in modulating aneurysm pathogenesis (Stefanadis et al., 1999; Xing et al., 2009; Detaint et al., 2010). In particular, estrogen showed a protective effect toward adverse cardiovascular manifestations in human and animal studies (Stefanadis et al., 1999; Gonzales et al., 2001; Martin-McNulty et al., 2003). Furthermore, the vasculoprotective effects of estrogen have been extensively documented in cardiovascular disease and include suppression of inflammatory cell recruitment, reduction of pro-inflammatory signaling, and preservation of vascular homeostasis following injury (Natoli et al., 2005; Boese et al., 2017; Saddic et al., 2023). In a recent study, Saddic et al. (2023) demonstrated that 17β-estradiol treatment in male MFS mice reduced TNFα-mediated NF-κB signaling, preserved vSMC function, and slowed aortic root dilation. These findings provide mechanistic evidence supporting a protective role of estrogen in TAA development, progression, and adverse outcomes.

Despite these advances, important knowledge gaps remain. Previous studies have largely focused on male animals, and the influence of altered estrogen levels on TAA progression and outcomes in females has not been investigated. Moreover, the contribution of estrogen to ECM remodeling and the biomechanical integrity of the aortic wall remains poorly understood. This gap is particularly significant because pathological ECM remodeling and changes in wall mechanical properties are fundamental determinants of aneurysm growth and the risk of aortic dissection or rupture (Weiss et al., 2023; Dwivedi et al., 2024; Dwivedi et al., 2025). Therefore, defining the role of estrogen in regulating aortic wall remodeling and stiffness in males and females is essential for understanding the mechanisms underlying sex-specific differences in TAA disease.

In this study, we investigated the effects of increased estrogen in male mice through an implanted pellet of 17β-estradiol and reduced estrogen in female mice through bilateral oophorectomy (OVX) (a postmenopausal-like condition) (Rodriguez-Landa, 2022) on TAA growth, ECM remodeling, and *in vivo* stiffness. We used *Fbn1^mgR/mgR^* mice, a well-established MFS model that reproduces the cardiovascular phenotypes characteristic of severe MFS in humans, including rapid aortic dilation and early death from cardiovascular complications (Pereira et al). Our findings demonstrate that estrogen slows aneurysmal dilation, prevents elastic fiber degradation in the ECM, reduces age-dependent stiffening, and extends lifespan in male and female mice.

## 2 Materials and methods

### 2.1 Mice

All animal use and experimental protocols were approved by the Washington University and University of Utah Institutional Animal Care and Use Committee and conform to the current NIH guidelines. We used a mouse model of MFS with low fibrillin-1 levels, *Fbn1^mgR/mgR^*, that mimics TAA manifestations of severe MFS in humans (Pereira et al). *Fbn1^mgR/mgR^* mice were maintained on a C57BL6J background and refreshed by backcrossing every three generations. After genotyping confirmation, *Fbn1^mgR/mgR^* mice were separated into male and female groups. The complete experimental procedure for this is illustrated in **Figure 1**.

**Figure 1:**
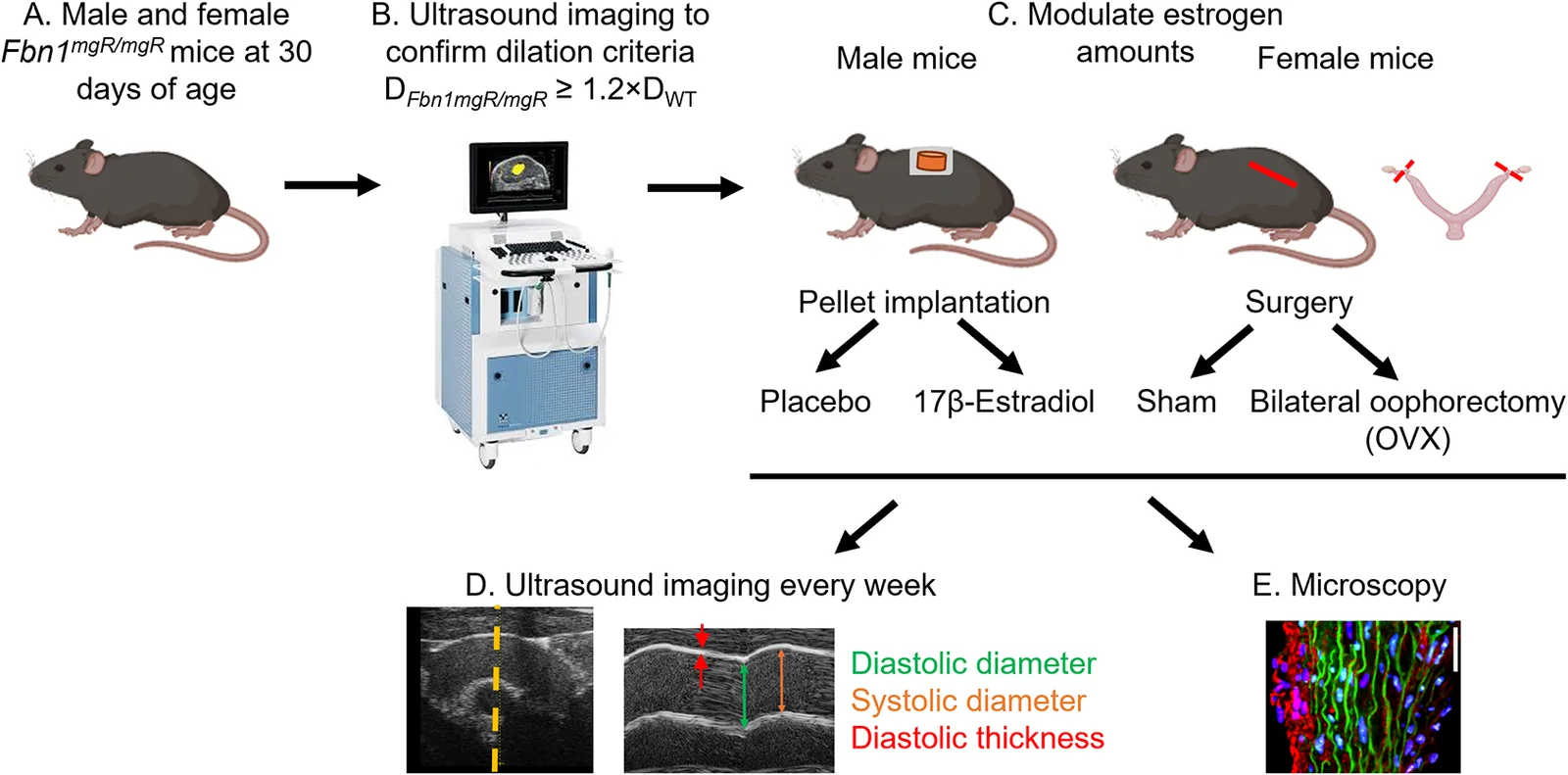
After genotyping **(A)**, TAA phenotype was confirmed by ultrasound imaging at 30 days of age **(B)**. *Fbn1^mgR/mgR^* mice with a diastolic ascending thoracic aortic (ATA) diameter ≥1.2-fold that of age-matched wild-type (WT) mice were enrolled in the study. Male mice received either implanted placebo or 17β-estradiol pellets, whereas female mice underwent either sham or bilateral ovariectomy (OVX) surgery **(C)**. Post-treatment ATA dimensions were monitored weekly by ultrasound until death **(D)**. Mice that died during the study were collected and the ATA was processed for microscopy, if possible **(E)**.

### 2.2 Inclusion criteria

Early TAA phenotype was confirmed by measuring the ascending thoracic aorta (ATA) inner diameter during diastole of *Fbn1^mgR/mgR^* male and female mice at 30 days of age using ultrasound imaging (see Section 2.5 for detailed imaging procedures). The included male and female mice had average diameters of 1.465 ± 0.166 and 1.456 ± 0.182 mm, respectively, which was 1.462 and 1.452-fold greater than age- and sex-matched wild type (WT) ATA diameters from our previous study (Dwivedi et al., 2024) and met the inclusion criteria (diastolic diameter of *Fbn1*^mgR/mgR^ ≥ 1.2-fold diastolic diameter WT). All mice were checked daily for survival. If mice died at any age during the study, the cause of death was confirmed through necropsy and the ATA was removed, fixed in 10% formalin and stored at 4°C for microscopy imaging (Section 2.6).

### 2.3 Procedures to modulate estrogen amounts

*Fbn1^mgR/mgR^* male mice were randomly assigned to receive either 17β-estradiol treatment or placebo control treatment at 30 days of age. *Fbn1*^*mgR/mgR*^ female mice were randomly assigned to undergo either bilateral ovariectomy (OVX) or sham surgery at 30 days of age (**Figure 1**). For both sexes, they were anesthetized with 2% isoflurane and the insertion/surgical sites were shaved and aseptically prepared using alternating applications of betadine and 100% ethanol at least three times. For male mice, a small (< 1 cm) dorsal, subcutaneous incision was made, and mice received either a 17β-estradiol pellet or a placebo pellet (0.25 mg/pellet, 60-day release; Innovative Research of America, Sarasota, FL). The incision was then closed using autoclips. For female mice, OVX was performed through bilateral flank incisions in a single surgical session. To minimize the risk of cross-contamination, a separate set of sterilized surgical instruments was used for each side, and standard aseptic surgical procedures were followed throughout, including appropriate personal protective equipment, sterile surgical gloves, and disinfection of the surgical platform. A small (3–5 mm) flank incision was made through the skin, subcutaneous tissue, and abdominal muscle wall. The ovary and uterine horn were exteriorized by gently elevating the associated fat pad. The ovary was removed using cautery, after which the uterine horn and fat pad were returned to the abdominal cavity. The muscle layer was sutured, and the skin was closed using autoclips. For sham-operated female mice, the same bilateral surgical procedure was followed as for OVX; however, the ovaries were exteriorized and immediately returned to the abdominal cavity without removal, followed by closure of the muscle and skin layers using the same procedure. The complete OVX procedure was completed within 30–40 minutes. Following surgery, all mice were monitored twice daily, in the morning and evening, for general health, postoperative recovery, and survival, and no signs of surgical-site infection were observed. Autoclips were removed 10 days after surgery. Body weight was recorded before treatment and subsequently monitored at weekly intervals throughout the study.

### 2.4 Immunoassays

Circulating estradiol levels were measured using a mouse estradiol rapid enzyme-linked immunosorbent assay (ELISA) kit (Cat. #EELR013, Invitrogen). Blood samples were collected via the submandibular route at 30, 60, and 90 days of age. At each time point, 4–5 drops of blood were collected according to the IACUC-approved protocol. Blood samples were kept at room temperature for 1 hour to allow clot formation, followed by centrifugation for serum separation. Serum analysis and estradiol measurements were performed according to the ELISA kit manufacturer’s instructions. Data were not obtained from all mice at each timepoint due to delays in starting blood collection, mortality before the scheduled blood collection time point, or insufficient serum volume for some of the collected blood samples. In addition, seven wells in the ELISA plate showed unreliable optical density readings close to zero and were therefore excluded from the analysis.

### 2.5 Cardiovascular ultrasound

*In vivo* ATA geometry of each mouse at 30 days of age and weekly post treatment up to a maximum of 90 days of age was measured using a high-resolution small animal ultrasound imaging system (Vevo 2100, FUJIFILM VisualSonics). Mice were anesthetized with 2% isoflurane through a nose cone, monitored by toe pinch, and kept warm. M-mode images of the ATA were collected in the central region between the aortic root and brachiocephalic artery. ATA inner diastolic diameter (*d_dia_*), systolic (*d_sys_*) diameter and diastolic thickness (*t_dia_*) were measured using post processing software (Vevo Lab, FUJIFILM VisualSonics). The ATA geometry measurement was performed every week until death of the mouse. Peterson’s modulus (*E_p_*), a measure of structural stiffness, and *in vivo* incremental Young’s modulus (*E_inc_*), a measure of material stiffness, were determined using Equations 1 and 2, respectively (Gosling and Budge, 2003). The age-matched pulse pressure (pp) values for male and female *Fbn1^mgR/mgR^* mice were taken from our previous study (Dwivedi et al., 2024).

(1) $$E_{p}=\frac{d_{dia}\times pp}{\left(d_{sys}-d_{dia}\right)}$$

(2) $$E_{inc}=\frac{d_{dia}^{2}\times pp}{2t_{dia}\left(d_{sys}-d_{dia}\right)}$$

### 2.6 Multiphoton and immunofluorescence imaging

For mice that died before the treatment endpoint of 90 days of age, the ATA was harvested within 12–14 h after death. The postmortem interval was recorded for each mouse and kept consistent across experimental groups. Because ECM proteins are relatively stable and vascular tissues retain histological integrity over short postmortem intervals (Huang et al., 2024), tissues collected within this window were included for structural ECM and α-SMA analyses. The ATA was rinsed in phosphate buffered saline (PBS) and fixed in 10% formalin overnight. After washing in PBS, the ATA was mounted in optimum cutting temperature (OCT) solution, cryosectioned (8–10 micrometer thickness), and mounted on a charged slide. Following the OCT wash, sections were either used for multiphoton (ECM) or immunofluorescence (α-SMA) imaging.

For multiphoton imaging, the ATA section was stained with DAPI (Invitrogen, P36935) for cell nuclei and imaged using a sp-8 DIVE multiphoton microscope (Leica) with a 40x objective. Collagen fibers, elastic fibers, and cell nuclei were excited at 880 nm and imaged using second harmonic generation (SHG) (emission, 420–460 nm), autofluorescence (emission, 495–540 nm), and DAPI (emission, 430–450 nm), respectively (Dwivedi et al., 2024; Dwivedi et al., 2025). The elastic fiber area fraction and fragmentation index were measured as the normalized area of elastic fibers and the number of elastic fiber end points normalized by average branch size using FIJI (ImageJ, NIH), as previously described (Dwivedi et al., 2024; Dwivedi et al., 2025).

Immunofluorescence imaging was performed for α-SMA quantification. Following PBS wash, sections were blocked in 10% bovine serum albumin (Thermo Scientific, J10857.18). Sections were then stained with conjugated αSMA-Cy3 monoclonal antibody (Millipore Sigma, C6198) (1:300) overnight at 4 °C. Following gentle rocking in PBS for 30 minutes, sections were sealed with anti-fade mounting medium with DAPI and a coverslip. Immunofluorescent images were acquired on an Olympus BX53 microscope with an DP80 dual-sensor CCD camera using a 20x magnification objective. All images were acquired at the same exposure settings for consistent visual comparison. Quantification of α-SMA amount (area fraction) analysis was performed using FIJI (White et al., 2024).

### 2.7 Statistical analysis

All statistical analyses were performed using RStudio and GraphPad Prism. Investigators were blinded to mouse groups during data analysis. Male and female datasets were imported separately and combined after standardizing treatment groups and age categories. Mouse identification was included as a random effect to account for repeated measurements obtained from the same animal over time. Longitudinal changes in the measured parameters were analyzed separately for male and female mice using linear mixed-effects models (LMMs; lme4 package), with treatment, age, and their interaction included as fixed effects. Analysis of variance (ANOVA) was performed on the fitted models to evaluate the significance of main effects and interactions. Estimated marginal means were calculated using the emmeans package, and pairwise treatment comparisons at each age were adjusted using the Holm method for sex-specific analyses. Diameter and thickness over time, between treatment groups, and between age points within groups were compared using Student’s *t*-tests, as appropriate. Longitudinal data are presented as mean ± standard error (SEM) with individual animal trajectories included. Bar graphs present mean ± standard deviation (SD) with individual mouse data shown.

The absolute, percentage, and baseline-adjusted diastolic and systolic ATA dilation rates were further evaluated from the cardiovascular ultrasound data. Absolute ATA dilation rate (mm/day) was estimated for each animal as the slope of ATA diameter versus age using all available longitudinal measurements from Day 30 until death or completion of follow-up. Percentage dilation rate (%/day) was calculated by normalizing the individual dilation slope to the corresponding Day-30 ATA diameter and multiplying by 100. Thus, all available measurements obtained before death contributed to the estimation of individual aortic dilation rates. Group differences in dilation rate were evaluated using ANCOVA, with the corresponding Day-30 ATA diameter included as a covariate to account for differences in baseline aortic size. For baseline adjustment, the overall mean Day-30 ATA diameter across animals included in the analysis was used as the common reference baseline. Each animal’s observed dilation rate was standardized to this reference baseline using the baseline-diameter coefficient estimated from the ANCOVA model, thereby accounting for differences in initial ATA diameter while retaining inter-animal variability. Baseline-adjusted estimated marginal mean dilation rates and corresponding 95% confidence intervals were then calculated at this common reference diameter, allowing treatment groups to be compared at an equivalent baseline aortic size. The group × baseline diameter interaction was evaluated to assess whether the relationship between baseline diameter and dilation rate differed among groups. The comparisons of unadjusted and baseline-adjusted dilation rates were performed between the treatment groups using Wilcoxon test. Serum estradiol levels were compared between treatment groups within each sex using two-way ANOVA.

Survival analysis was performed using Kaplan–Meier curves with log-rank tests. Pairwise comparisons were adjusted using the Holm method, and Cox proportional hazards regression was performed to estimate hazard ratios (HRs) for the comparisons of placebo versus 17β-estradiol-treated male mice and sham versus OVX female mice. Mice sacrificed at 90 days of age for a separate study were excluded from survival analyses but were included in geometry and *in vivo* stiffness analyses at earlier time points.

Comparisons of elastic fiber fragmentation, elastic fiber fraction, and α-SMA amount between treatment groups within each sex were performed using unpaired Welch’s *t*-tests. A two-sided p< 0.05 was considered statistically significant for all analyses.

## 3 Results

### 3.1 Circulating estradiol levels

Circulating estradiol levels in the treatment groups of male and female mice are presented in **Figure 2**. In male mice, following 17β-estradiol treatment at 30 days, circulating estradiol concentrations increased significantly with age. At 60 and 90 days of age, 17β-estradiol-treated male mice showed significantly higher estradiol concentrations than placebo-treated mice (**Figure 2A**). Placebo-treated male mice also showed a significant decrease in estradiol concentration at 90 days compared with 30 days of age. In female mice, following OVX at 30 days of age, the mice showed a significant decrease in circulating estradiol concentrations. At 60 and 90 days of age, estradiol concentrations were significantly lower in OVX mice than in sham-operated mice (**Figure 2B**). A significant decrease in estradiol concentration was also observed in sham-operated female mice between 30 and 90 days of age.

**Figure 2:**
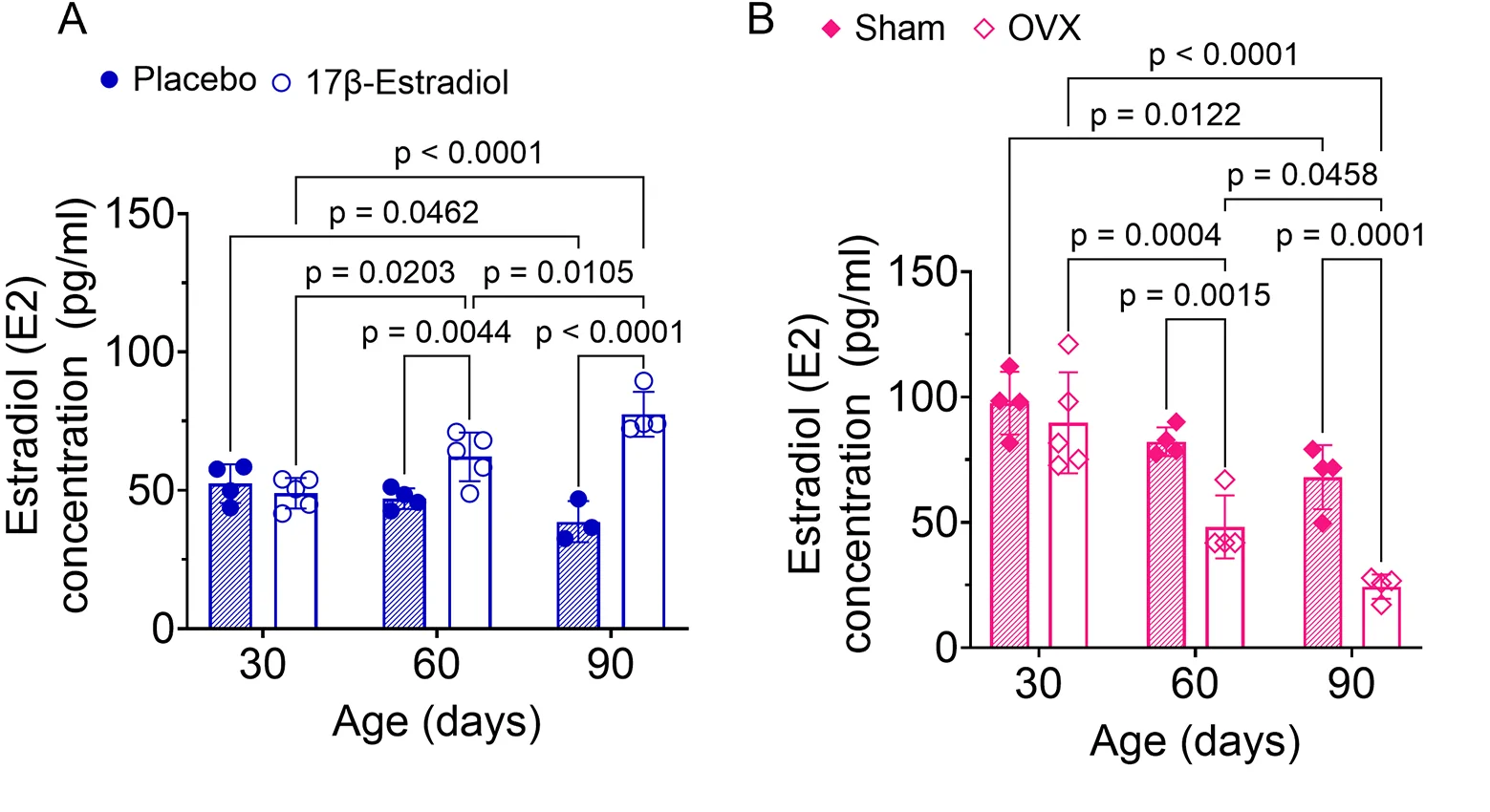
Circulating estradiol concentrations for **(A)** Placebo- and 17β-Estradiol-treated male mice and **(B)** Sham and OVX female mice. Comparisons at 30, 60 and 90 days of age are shown. For estradiol measurements, a total of 18 *Fbn1^mgR/mgR^* mice were included, comprising Placebo-treated males (n = 4), 17β-Estradiol-treated males (n = 5), Sham females (n = 4), and OVX females (n = 5). Significant p-values are shown.

### 3.2 Mouse body weight and survival

Body weight did not differ significantly between placebo- and 17β-estradiol-treated male mice or between sham and OVX female mice at 30, 45, 60, 75, or 90 days of age, indicating that hormonal interventions did not affect overall body weight during the study period (**Figures 3A, B**).

**Figure 3:**
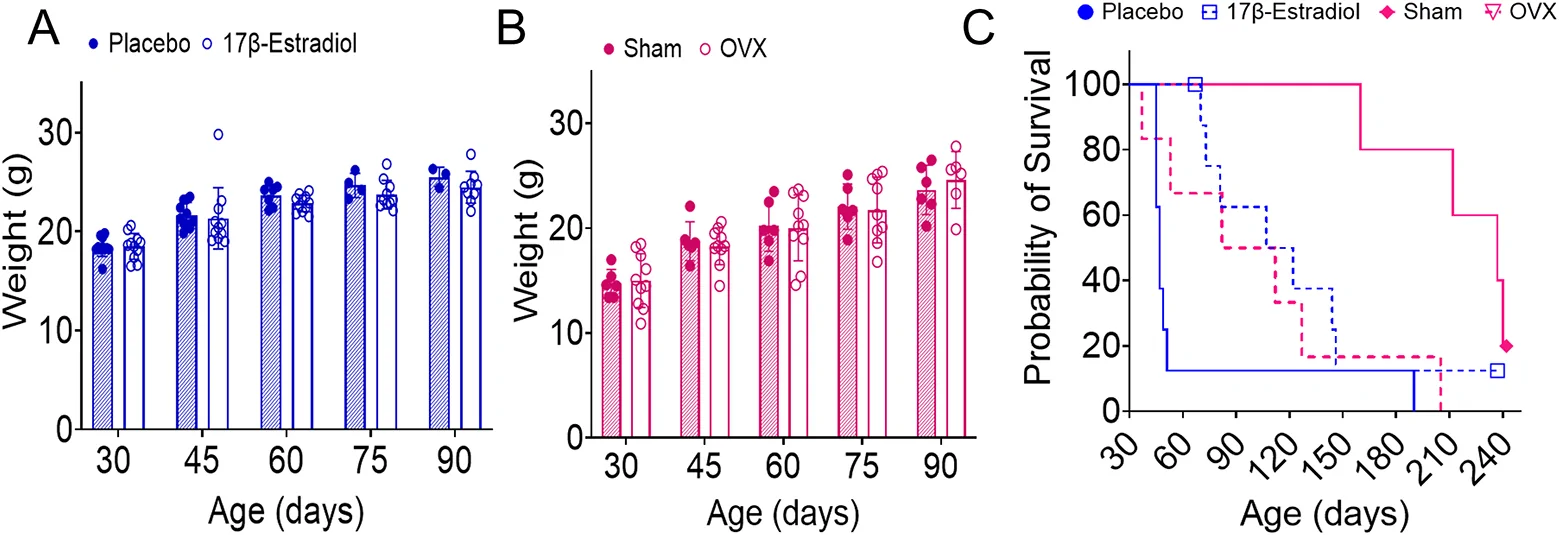
Body weight comparisons between **(A)** Placebo- and 17β-Estradiol-treated male mice and **(B)** Sham and OVX female mice. **(C)** Survival curve of Placebo and 17β-Estradiol-treated male mice and Sham and OVX female mice. The survival differed significantly among the four groups (log-rank test, χ^2^ = 17.21, *p* = 0.0006) with hazard ratio: 0.28 (Placebo versus 17β-Estradiol-treated male mice) and 14.66 (Sham versus OVX female mice). For survival analysis, a total of 31 *Fbn1^mgR/mgR^* mice were included, comprising Placebo males (n = 9), 17β-Estradiol-treated males (n = 9), Sham females (n = 5), and OVX females (n = 8).

The survival curves for individual *Fbn1^mgR/mgR^* mice following treatments are shown in **Figure 3C**. In male mice, 17β-estradiol treatment significantly prolonged survival compared with placebo treatment, increasing the median survival from 47 days to 122 days. In female mice, bilateral OVX significantly accelerated mortality compared with sham female mice, reducing the median survival from 224 days to 90 days. Overall survival differed significantly among the four groups (log-rank test, χ^2^ = 17.21, p = 0.0006). Cox proportional hazards analysis showed that 17β-estradiol treatment significantly reduced the risk of death in male mice compared with placebo treatment (hazard ratio = 0.28, p = 0.018), whereas OVX significantly increased the risk of death in female mice compared with sham surgery (hazard ratio = 14.66, p = 0.014). Survival of the placebo-treated males and sham females was similar to our previous work with untreated *Fbn1^mgR/mgR^* mice (Dwivedi et al., 2024).

### 3.3 ATA geometry

**Figure 4** shows the individual trajectories and group mean ± SEM of ATA diastolic diameter, systolic diameter, and diastolic wall thickness over time measured from ultrasound imaging. The diastolic and systolic ATA diameter values for each individual mouse at 30, 45, 60, 75, and 90 days of age, along with the corresponding mean and standard deviation (SD) at each time point, are provided in **Supplementary Tables S1**–**S8**. Placebo-treated male mice and OVX female mice exhibited larger increases in ATA diameter over time compared with 17β-estradiol-treated male mice and sham female mice, respectively. Note that the individual measurements end with mouse death, so the average curves are biased toward mice that survived until 90 days of age. The linear mixed effects with two-way ANOVA analysis for diastolic diameter (**Figures 4A, B**) showed that age was a significant factor in both male and female mice (male: *F* = 27.48, p< 0.0001; female: *F* = 34.40, p< 0.0001). In addition, estrogen treatment also had a significant effect in both sexes (male: *F* = 9.78, p = 0.0051; female: *F* = 11.58, p = 0.0035). Importantly, a significant treatment and age interaction was observed in both male (*F* = 5.03, p = 0.0014) and female mice (*F* = 9.87, p< 0.0001), demonstrating that the expected changes in diameter with age due to growth and TAA progression were modulated by estrogen treatment. For male mice systolic diameter (**Figure 4C**) was significantly affected by age (*F* = 26.80, p< 0.0001), and age and treatment interaction (*F* = 3.91, p = 0.0072), while for female mice, systolic diameter (**Figure 4D**) was significantly affected by age (*F* = 26.80, p< 0.0001), treatment (*F* = 7.13, p = 0.01664) and their interactions (*F* = 3.919, p = 0.0072). The lack of significance of treatment for systolic diameter in male mice is likely due to the lower numbers of mice at the higher ages due to mouse death in the male placebo group. ATA wall thickness was not influenced by treatment and only showed age dependent variation for both sexes (for male, *F* = 5.716, p = 0.00048; for female, *F* = 4.041, p = 0.0063) (**Figures 4E, F**).

**Figure 4:**
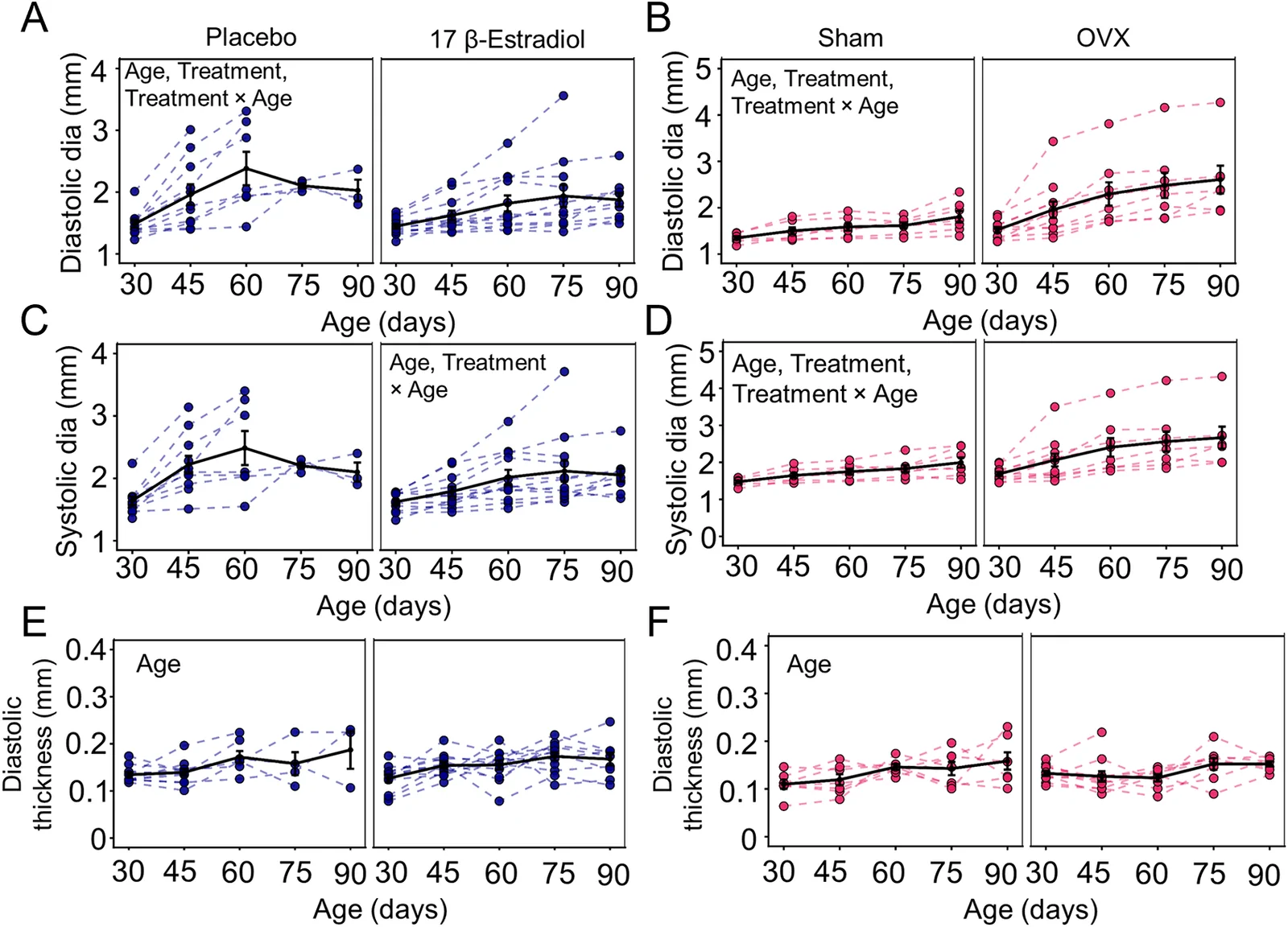
Longitudinal changes in ascending thoracic aortic (ATA) geometry for individual mice together with mean ± SEM for each group. Individual curves end at the last measurement before mouse death or 90 days of age, whichever is greater. Diastolic diameter is shown for **(A)** Placebo- and 17β-Estradiol-treated male mice and **(B)** Sham and OVX female mice. Systolic diameter is shown for **(C)** Placebo- and 17β-Estradiol-treated male mice and **(D)** Sham and OVX female mice. Diastolic thickness is shown for **(E)** Placebo- and 17β-Estradiol-treated male mice and **(F)** Sham and OVX female mice. Significant fixed effects of Age, Treatment, and their interaction (Age × Treatment) determined using linear mixed-effects models followed by two-way ANOVA are shown at the top of the panels. A total of 40 *Fbn1^mgR/mgR^* mice were included, comprising Placebo-treated males (n = 11), 17β-Estradiol-treated males (n = 11), Sham females (n = 7), and OVX females (n = 11).

Average (ATA) geometric parameters are shown for each group at 30, 45, and 60 days of age in **Figure 5** to show comparisons between treatment groups (placebo vs. 17β-estradiol and sham vs. OVX). Pairwise analyses were limited to a maximum of 60 days of age because approximately 90% of placebo-treated male mice died after 60 days, resulting in insufficient statistical power for reliable comparisons at older ages. At 30 days of age, no significant differences in ATA diastolic or systolic diameter were observed between treatment groups in either sex, indicating comparable baseline TAA severity prior to intervention. By 60 days of age, placebo-treated male mice exhibited significantly greater diastolic and systolic diameters than 17β-estradiol-treated mice (**Figures 5A, B**); however, at 45 days of age, only the systolic diameter was significantly greater in placebo-treated mice. In female mice, a significant difference between sham and OVX groups emerged at 60 days (**Figures 5C, D**). ATA diameter dilation of the placebo-treated males and sham females between 30 to 60 days of age was similar to our previous work with untreated *Fbn1^mgR/mgR^* mice (Dwivedi et al., 2024).

**Figure 5:**
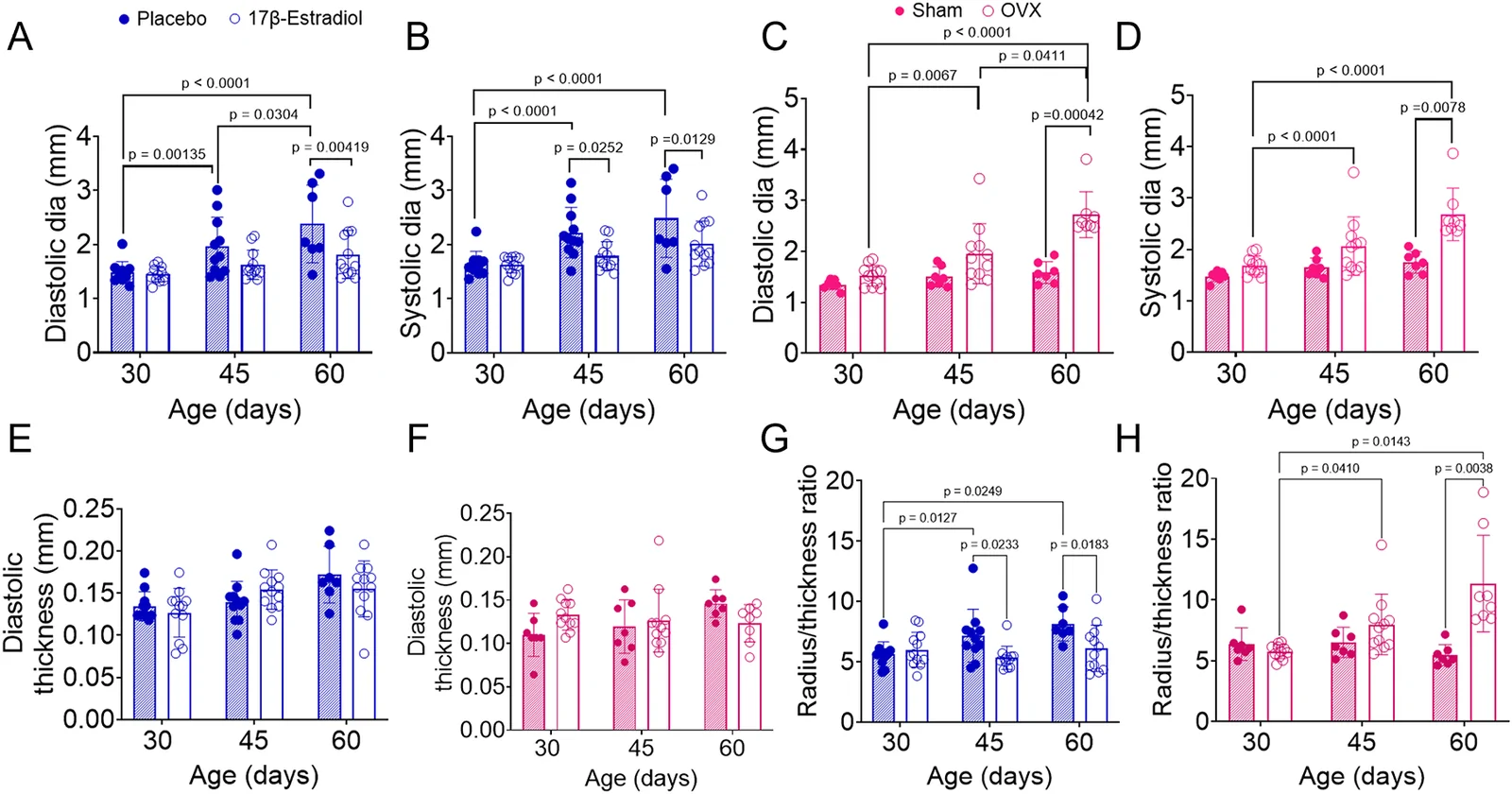
Pairwise comparisons of ascending thoracic aortic (ATA) geometry. Placebo versus 17β-Estradiol treated male mice for **(A)** diastolic and **(B)** systolic diameter and Sham versus OVX female mice for **(C)** diastolic and **(D)** systolic diameter. Placebo versus 17β-Estradiol treated male mice for **(E)** diastolic thickness and Sham versus OVX female mice for **(F)** diastolic thickness. Placebo versus 17β-Estradiol treated male mice for **(G)** diastolic radius to thickness ratio and Sham versus OVX female mice for **(H)** diastolic radius to thickness ratio. Comparisons at 30, 45, and 60 days of age are shown. Older ages were not included due to limited power for Placebo-treated males and OVX females due to mouse death. A total of 40 *Fbn1^mgR/mgR^* mice were included, comprising Placebo-treated males (n = 11), 17β-Estradiol-treated males (n = 11), Sham females (n = 7), and OVX females (n = 11). Significant p-values are shown.

Longitudinal comparisons within each treatment group showed no significant difference in ATA dilation between 30, 45, and 60 days for 17β-estradiol-treated male and sham female mice. In contrast, placebo-treated male mice and OVX female mice displayed progressive TAA enlargement, with significant increases in diastolic diameter between 30 and 45 days, 30 and 60 days, and 45 and 60 days. Similarly, systolic diameter increased significantly between 30 and 45 days and between 30 and 60 days in these groups. No significant differences between treatments or ages were observed for ATA wall thickness (**Figures 5E, F**). These results indicate the protective role of estrogen toward TAA progression in male and female *Fbn1^mgR/mgR^* mice.

To further evaluate aortic wall remodeling in TAA progression, we calculated the diastolic radius-to-thickness ratio. Placebo-treated male mice exhibited significantly greater diastolic radius-to-thickness ratios than 17β-estradiol-treated male mice at 45 and 60 days of age (**Figure 5G**), whereas OVX female mice showed significantly higher values than sham-operated mice at 60 days (**Figure 5H**). Furthermore, no significant differences in the diastolic radius-to-thickness ratio were observed among the 30-, 45-, and 60-day age groups in 17β-estradiol-treated male or sham-operated female mice. In contrast, placebo-treated male and OVX female mice exhibited significant increases in this ratio between 30 and 45 days and between 30 and 60 days of age.

We further calculated the absolute and percentage endpoint dilation rates for both diastolic and systolic ATA configurations for each mouse, along with the corresponding baseline-adjusted dilation rates, adjusted to a common reference based on the day-30 baseline ATA diameters. The mouse-specific values of absolute, percentage, and baseline-adjusted dilation rates, along with the corresponding adjusted baseline diameters for both diastolic and systolic ATA configurations, are provided in **Supplementary Tables S9**–**S16**. We observed a significant effect of estradiol status on ATA dilation rate. For the diastolic configuration, the unadjusted absolute dilation rate was significantly lower in 17β-estradiol-treated male mice than in placebo-treated males (p = 0.0084) and significantly greater in OVX females than in sham females (p = 0.0337) (**Figure 6A**). Following adjustment for Day-30 baseline ATA diameter, the estimated dilation rate remained significantly lower in 17β-estradiol-treated males than in placebo-treated males (0.0126 vs. 0.0294 mm/day, Tukey-adjusted p = 0.0023) and significantly higher in OVX than in sham females (0.0253 vs. 0.0086 mm/day, Tukey-adjusted p = 0.0126) (**Figure 6B**).

**Figure 6:**
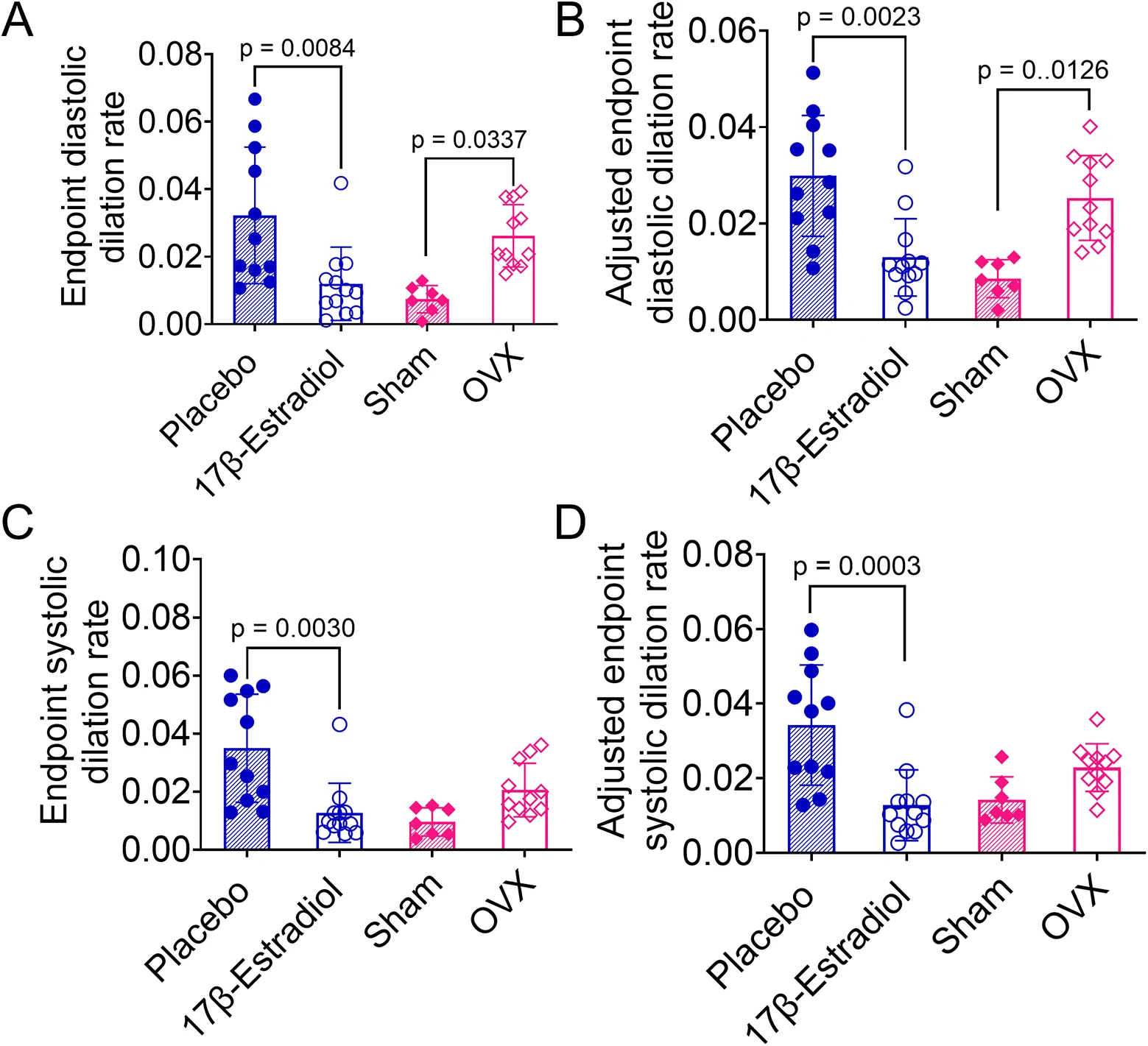
Pairwise comparisons of ascending thoracic aortic (ATA) endpoint dilation rate. Absolute **(A, C)** and 30-day baseline adjusted **(B, D)** diastolic **(A, B)** and systolic **(C, D)** dilation rates are shown. A total of 40 *Fbn1^mgR/mgR^* mice were included, comprising Placebo-treated males (n = 11), 17β-Estradiol-treated males (n = 11), Sham females (n = 7), and OVX females (n = 11). Significant p-values are shown.

For the systolic configuration, the unadjusted dilation rate was significantly lower in 17β-estradiol-treated males than in placebo-treated males (p = 0.0030), whereas the difference between OVX and sham females was not significant (**Figure 6C**). After adjustment for Day-30 systolic ATA diameter, the dilation rate remained significantly lower in 17β-estradiol-treated males than in placebo-treated males (0.0128 vs. 0.0342 mm/day, Tukey-adjusted p = 0.0003). The adjusted dilation rate was numerically higher in OVX females than in sham females (0.0183 vs. 0.0142 mm/day), but this difference was not significant (Tukey-adjusted p = 0.8924) (**Figure 6D**).

### 3.4 *In vivo* ATA wall stiffness

**Figure 7** illustrates the individual trajectories and group mean ± SEM of the structural and material stiffness of the ATA wall measured from ultrasound imaging for each group. Structural stiffness is calculated from Eqn. 1 and incorporates material and geometric effects, including wall thickness, whereas material stiffness is calculated from Eqn. 2 and is independent of geometry. Placebo male and OVX female mice exhibited a progressive increase in structural (**Figures 7A, B**) and material wall stiffness (**Figures 7C, D**) with age. In contrast, 17β-estradiol-treated male mice and sham female mice showed minimal changes in these biomechanical parameters with age. For both male and female mice, the change in ATA wall structural stiffness was significantly affected by age (male, *F* = 12.51, p< 0.0001; female, *F* = 7.62, p< 0.0001), treatment (male, *F* = 22.84, p< 0.0001; female, *F* = 13.98, p = 0.0022) and their interaction (male, *F* = 6.609, p = 0.00016; female, *F* = 7.85, p< 0.0001). Similar trends were found for wall material stiffness for both male and female mice, which was significantly affected by age (male, *F* = 15.21, p< 0.0001; female, *F* = 5.10, p = 0.0014), treatment (male, *F* = 21.13, p = 0.00013; female, *F* = 7.299, p = 0.015) and their interaction (male, *F* = 7.813, p< 0.00010; female, *F* = 5.134, p = 0.0013).

**Figure 7:**
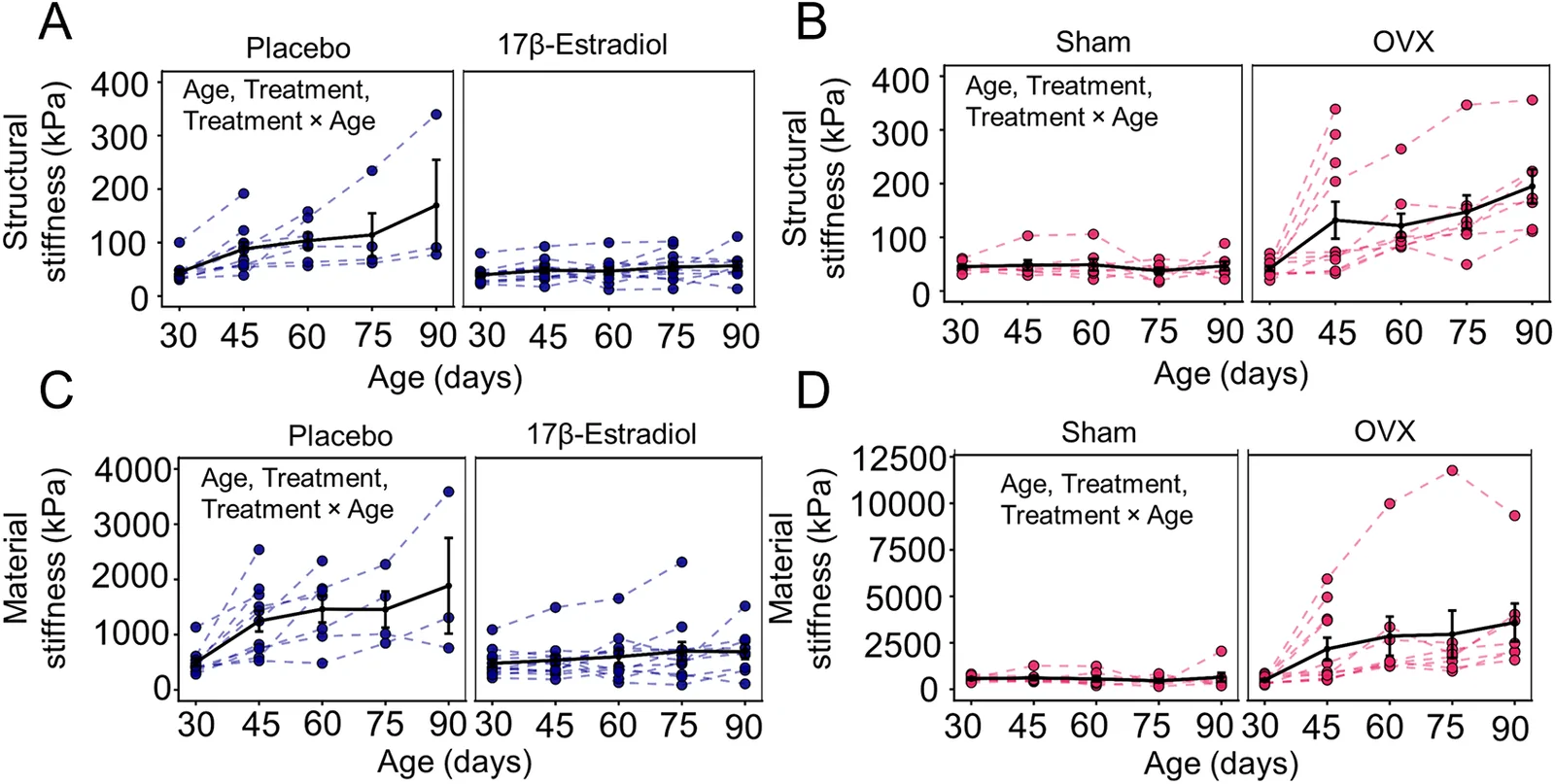
Longitudinal changes in ascending thoracic aortic (ATA) wall stiffness for individual mice together with mean ± SEM for each group. Individual curves end at the last measurement before mouse death or 90 days of age, whichever is greater. Structural wall stiffness is shown for **(A)** Placebo- and 17β-Estradiol-treated male mice and **(B)** Sham and OVX female mice. Material wall stiffness is shown for **(C)** Placebo- and 17β-Estradiol-treated male mice and **(D)** Sham and OVX female mice. Significant fixed effects of Age, Treatment, and their interaction (Age × Treatment) determined using linear mixed-effects models followed by two-way ANOVA are shown at the top of the panels. A total of 40 *Fbn1^mgR/mgR^* mice were included, comprising Placebo-treated males (n = 11), 17β-Estradiol-treated males (n = 11), Sham females (n = 7), and OVX females (n = 11).

To further characterize the effects of estrogen modulation on wall stiffness, pairwise comparisons of structural and material wall stiffness were performed between treatment groups at 30, 45, and 60 days of age and among age groups within each treatment (**Figure 8**). Structural (**Figures 8A, B**) and material stiffness (**Figures 8C, D**) of the ATA wall were significantly greater in placebo-treated male mice than in 17β-estradiol-treated male mice and in OVX female mice than in sham female mice at 45 and 60 days of age, demonstrating that estrogen deficiency is associated with accelerated aortic wall stiffening in male and female *Fbn1^mgR/mgR^* mice. There were no significant changes in structural or material stiffness between 30, 45, and 60 days in 17β-estradiol-treated male or sham female mice, indicating preserved wall stiffness over time in *Fbn1^mgR/mgR^* mice with high estrogen levels. In contrast, placebo-treated male and OVX female mice exhibited significant increases in both structural and material stiffness between 30 and 45 days and between 30- and 60-days indicating age-dependent aortic stiffening in *Fbn1^mgR/mgR^* mice with low estrogen levels. Changes in the *in vivo* wall stiffness of the placebo-treated males and sham females between 30 to 60 days of age were similar to our previous work with untreated *Fbn1^mgR/mgR^* mice (Dwivedi et al., 2024).

**Figure 8:**
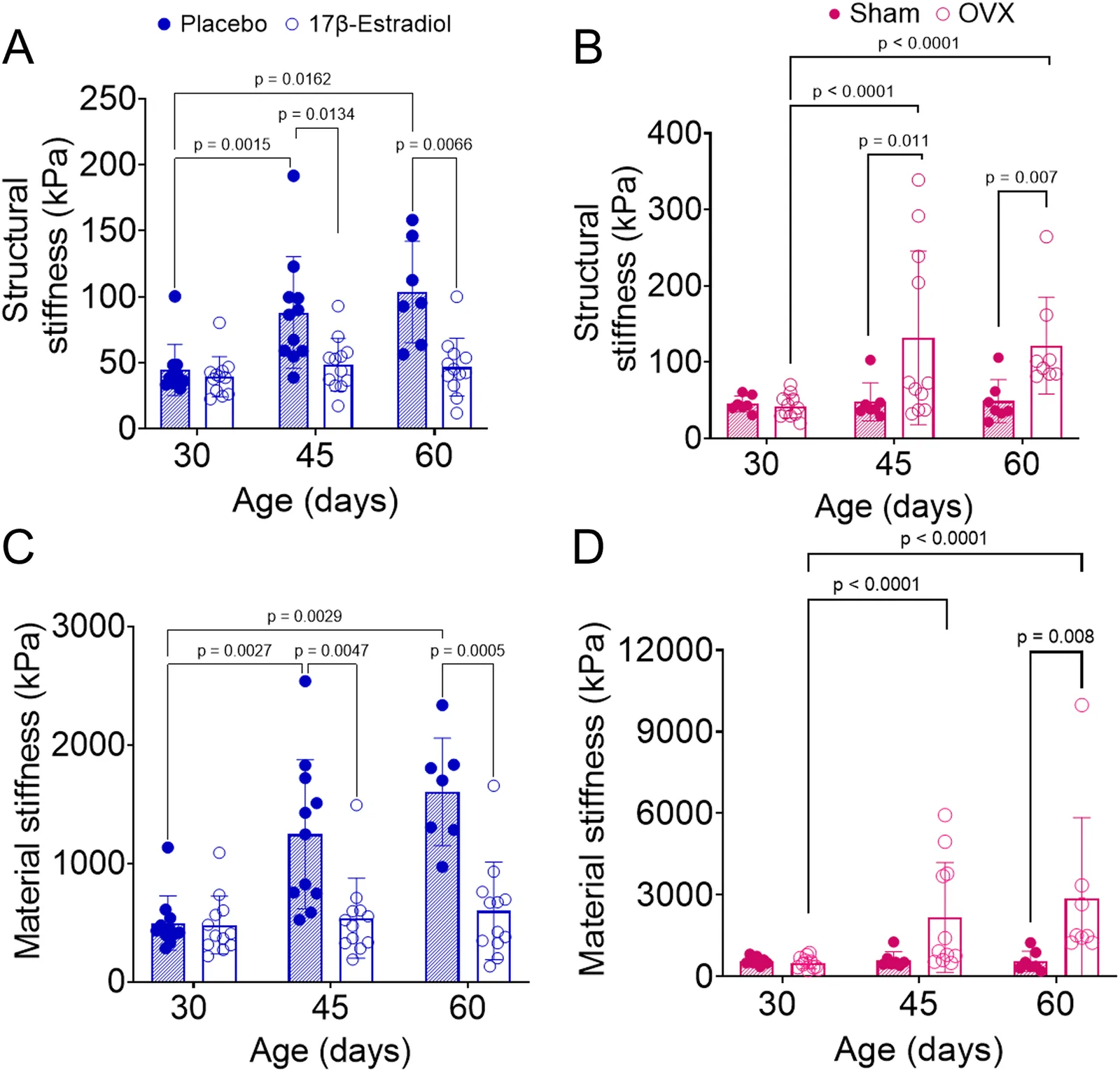
Pairwise comparisons of ascending thoracic aortic (ATA) wall stiffness. Structural stiffness for Placebo versus 17β-Estradiol treated male mice **(A)** and Sham versus OVX female mice **(B)**. Material stiffness for Placebo versus 17β-Estradiol treated male mice **(C)** and Sham versus OVX female mice **(D)**. Comparisons at 30, 45, and 60 days of age are shown. Older ages were not included due to limited power for Placebo-treated males and OVX females due to mouse death. A total of 40 *Fbn1^mgR/mgR^* mice were included, comprising Placebo-treated males (n = 11), 17β-Estradiol-treated males (n = 11), Sham females (n = 7), and OVX females (n = 11). Significant p-values are shown.

### 3.5 ECM remodeling and vSMC amount

**Figures 9A–D** show representative cross-sectional multiphoton images of the ATA collected after mouse death. Qualitative assessment revealed marked elastic fiber fragmentation, reduced elastic lamellar organization, and decreased cellularity in placebo-treated male mice and OVX female mice. In contrast, ATA sections from 17β-estradiol-treated male mice and sham-operated female mice exhibited well-preserved elastic fibers with continuous elastic lamellae and greater cellular density, indicating maintenance of medial wall architecture. Furthermore, OVX female mice displayed prominent collagen accumulation within the medial layer, suggesting enhanced ECM remodeling associated with estrogen depletion.

**Figure 9:**
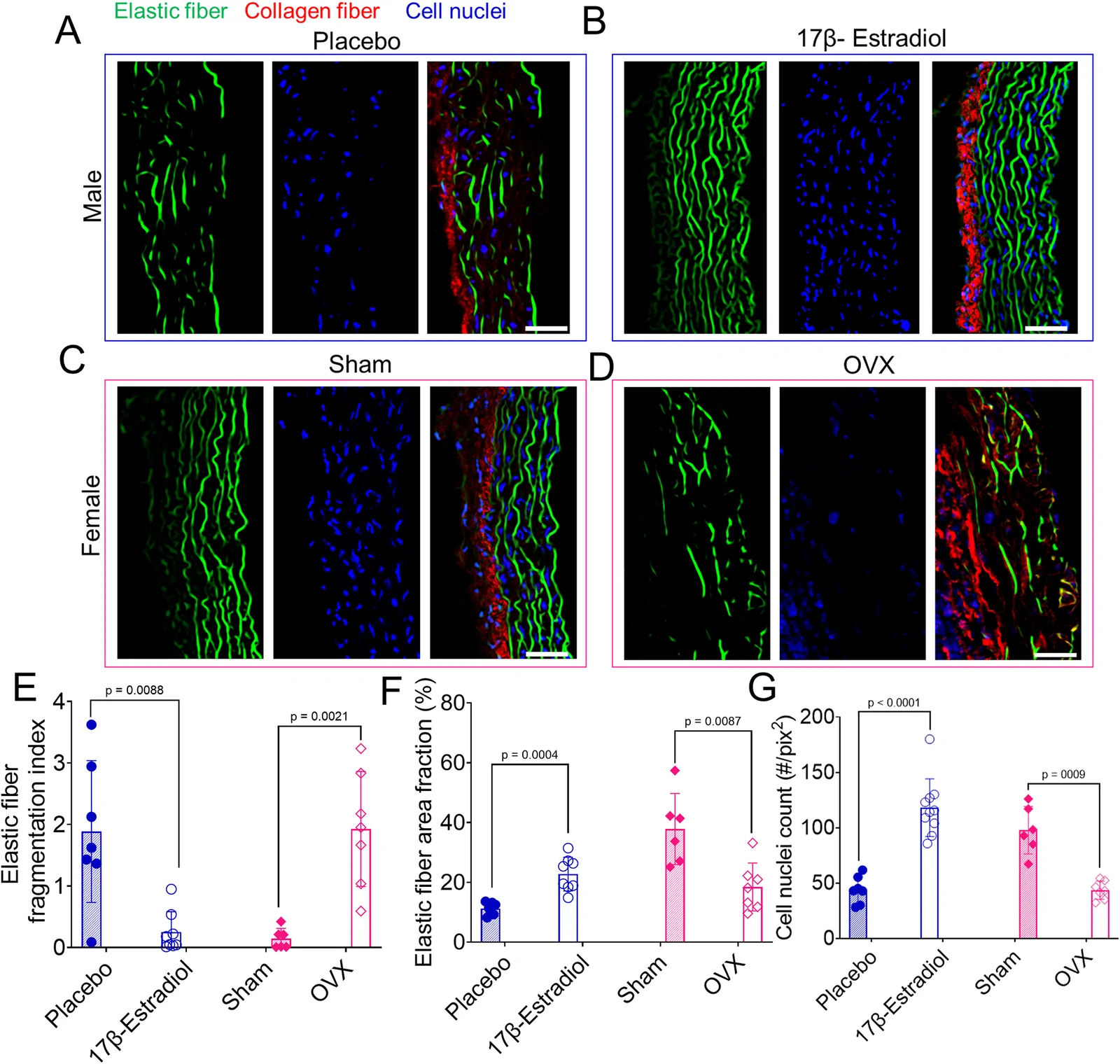
Representative cross-sectional multiphoton images of the ascending thoracic aortic (ATA) wall from **(A)** Placebo-treated male, **(B)** 17β-Estradiol-treated male, **(C)** Sham female, and **(D)** OVX female mice, collected at the time of death. Elastic fibers are green, collagen fibers are red, and cell nuclei are blue. Scale bar = 50 μm. Quantification of extracellular matrix (ECM) constituents from multiphoton images indicates **(E)** elastic fiber fragmentation index, **(F)** elastic fiber area fraction, and **(G)** cell nuclei count for Placebo and 17β-Estradiol-treated male mice and Sham and OVX female mice. A total of 28 *Fbn1^mgR/mgR^* mice were included for elastic fiber fragmentation and fraction analysis, comprising Placebo-treated males (n = 7), 17β-Estradiol-treated males (n = 8), Sham females (n = 6), and OVX females (n =7). A total of 30 *Fbn1^mgR/mgR^* mice were included for nuclei count analysis, comprising Placebo-treated males (n = 7), 17β-Estradiol-treated males (n = 10), Sham females (n = 6), and OVX females (n =7). Significant p-values are shown.

To quantitatively evaluate these structural changes, elastic fiber fragmentation was assessed by measuring the number of elastic fiber endpoints, elastic fiber amount was determined as the percentage of medial area occupied by elastin fluorescence, and cell nuclei were quantified by counting nuclei within the medial area (**Figures 9E–G**). Compared with 17β-estradiol-treated male mice and sham-operated female mice, placebo-treated male and OVX female mice respectively exhibited significantly increased elastic fiber fragmentation (**Figure 9E**), reduced elastic fiber amount (**Figure 9F**), and decreased medial cell nuclei density (**Figure 9G**). These findings demonstrate that estrogen limits ECM degeneration and cell dropout during TAA progression. The trends for sex differences in elastic fiber fragmentation and area fraction and cell nuclei counts in the placebo-treated males and sham females were similar to our previous work with untreated *Fbn1^mgR/mgR^* mice (Dwivedi et al., 2024).

Immunohistochemical staining demonstrated significantly reduced α-SMA protein levels in placebo-treated male and OVX female mice compared with 17β-estradiol-treated male and sham-operated female mice, respectively (**Figure 10**). As α-SMA is a key contractile protein expressed by vSMCs, these findings indicate that estrogen preserves the contractile phenotype of vSMCs during TAA progression.

**Figure 10:**
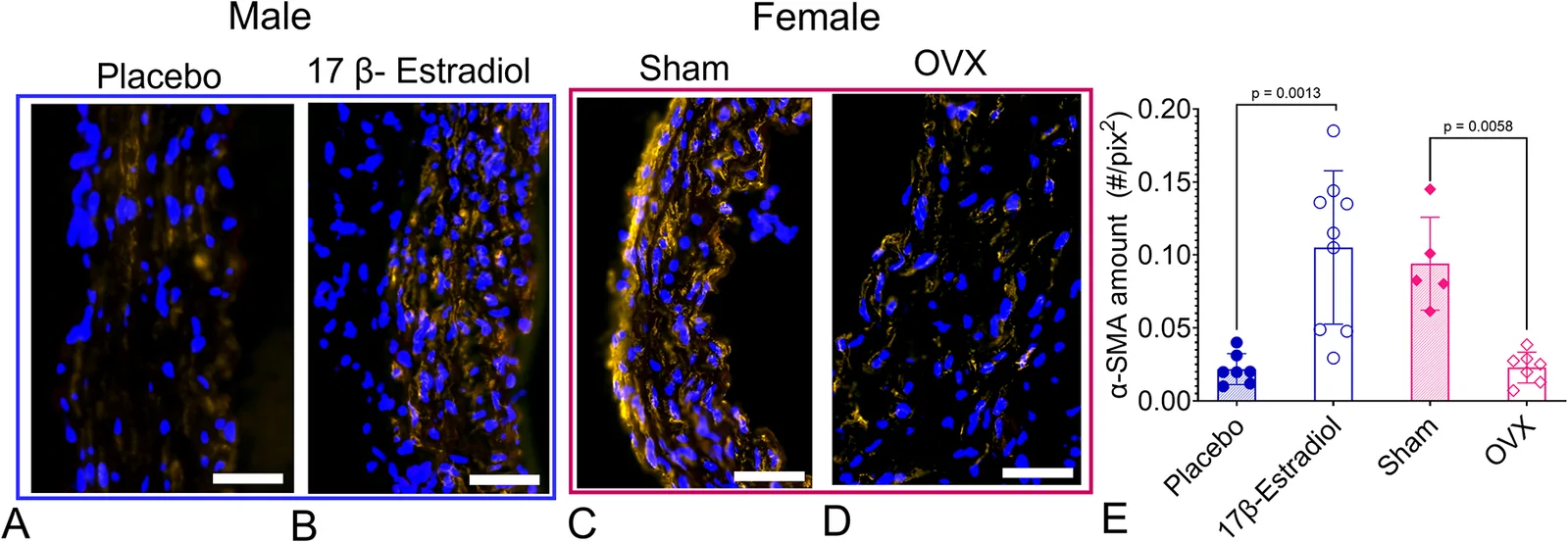
Representative immunofluorescence images of the ascending thoracic aortic (ATA) wall from **(A)** Placebo-treated male, **(B)** 17β-Estradiol-treated male, **(C)** Sham female, and **(D)** OVX female mice, collected at the time of death. α-SMA is yellow/orange and cell nuclei are blue. Scale bar = 50 μm. Quantification of α-SMA amount **(E)** for Placebo and 17β-Estradiol-treated male mice and Sham and OVX female mice. A total of 28 *Fbn1^mgR/mgR^* mice were included, comprising Placebo-treated males (n =7), 17β-Estradiol-treated males (n = 9), Sham females (n = 5) and OVX females (n = 7).

## 4 Discussion

In this study, we investigated the role of estrogen in MFS associated TAA development, progression, and outcomes. Using the severe MFS mouse model (*Fbn1^mgR/mgR^*), we included both male and female mice to evaluate the sex-specific effects of estrogen modulation. Male *Fbn1^mgR/mgR^* mice received either 17β-estradiol or placebo pellets and female *Fbn1^mgR/mgR^* mice underwent either bilateral OVX or sham surgery. Bilateral OVX is a well-established experimental model of the human postmenopausal state that mimics the loss of ovarian function and associated decline in endogenous estrogen production (Rodriguez-Landa, 2022). Following treatment, we monitored survival, assessed ATA dilation and wall stiffness over time, and visualized ECM remodeling and α-SMA protein amounts in the ATA. The primary objective of this study was to investigate mechanisms for sexual dimorphism in TAA associated with MFS and build upon our previous findings that female *Fbn1^mgR/mgR^* mice exhibited preserved wall mechanics and ECM integrity, slower aneurysm progression, and improved survival compared with male mice (Dwivedi et al., 2024; Dwivedi et al., 2025).

### 4.1 Previous evidence of sex dependency and estrogen protective effects in TAA

Despite the equal prevalence of MFS in males and females, both clinical and animal studies have demonstrated marked sex differences in TAA development, progression, and adverse aortic events, including dissection and rupture (Detaint et al., 2010; Roman et al., 2017; Chung et al., 2020; Gharraee et al., 2022; Dwivedi et al., 2024). Adult male patients with MFS exhibit a higher incidence of aortic root dilation, prophylactic aortic root replacement, and aortic dissection than age-matched female patients (Detaint et al., 2010; Roman et al., 2017). In contrast, female patients generally display a slower disease progression during early adulthood but experience an increased risk of severe TAA outcomes, including aortic dissection, postoperative complications, and mortality, later in life (Crousillat et al., 2023; Crosier et al., 2024). Consistent with clinical observations, animal studies have also demonstrated a protective response of female sex against MFS-associated TAA progression. Gharraee et al. (Gharraee et al., 2022) used the mild MFS mouse model (*Fbn1^C1041G/+^*) and reported that female mice exhibited reduced aortic dilation, preserved ECM organization, and maintenance of the vSMC contractile phenotype compared with male mice. Similarly, Jimenez-Altay et al. (Jimenez-Altayo et al., 2017) used the *Fbn1^C1041G/+^* MFS mouse model and observed that female mice displayed preserved ATA wall contractility and reduced elastic fiber fragmentation compared to males. Our previous study using the *Fbn1^mgR/mgR^* mouse model further demonstrated that female mice maintain favorable wall biomechanics and ECM integrity, resulting in slower TAA progression and prolonged survival compared with male mice (Dwivedi et al., 2024; Dwivedi et al., 2025; Dwivedi et al., 2026). Collectively, these findings consistently indicate that female sex either confers protection against TAA progression or delays the onset of adverse aortic events.

The underlying mechanisms responsible for this sexual dimorphism remain incompletely understood; however, sex hormones, particularly estrogen, are likely to play a critical role (Laser et al., 2014; Boese et al., 2017; Qi et al., 2020). A recent study (Saddic et al., 2023) using *Fbn1^C1041G/+^* mice investigated the effects of 17β-estradiol treatment on TAA progression and demonstrated that estrogen significantly attenuated aortic root dilation and reduced the incidence of aortic rupture when mice were infused with Angiotensin II. Mechanistically, the authors reported that 17β-estradiol preserved vSMC contractile phenotype by suppressing pro-inflammatory TNFα-mediated NF-κB signaling. However, this study was limited to male mice and therefore did not address the role of endogenous estrogen depletion or the effects of estrogen modulation in female mice.

### 4.2 Estrogen affects mouse survival

*Fbn1^mgR/mgR^* mice died in this study due to aortic wall rupture mostly at the site of TAA, consistent with previous results (Pereira et al., 1999). After death of each mouse, we confirmed the cause of death through necropsy. The survival curves demonstrate a clear separation between estrogen-modulated and control groups in both sexes. In male mice, 17β-estradiol treatment significantly prolonged survival compared with placebo-treated mice, whereas estrogen depletion by bilateral OVX noticeably reduced survival in female mice compared with sham-operated controls. Notably, increasing estrogen levels in male mice shifted the survival profile toward that of sham-operated female mice, while estrogen depletion in females resulted in a survival pattern resembling placebo-treated male mice. The mice that died during the study showed blood in the chest cavity and signs of wall rupture between the aortic root and arch (**Supplementary Figure 1S**). Increased survival of 17β-estradiol treated male mice and decreased survival of OVX female mice indicates that estrogen is a major determinant of TAA progression and rupture susceptibility in the severe MFS mouse model and is consistent with our observed reductions in aortic dilation and stiffness following estrogen treatment.

### 4.3 Estrogen prevents aortic dilation and geometric remodeling

Our findings demonstrate that 17β-estradiol treatment markedly attenuates ATA dilation and rate of dilation in male *Fbn1^mgR/mgR^* mice, as evidenced by the minimal increase in *in vivo* aortic diameter and the significantly slower dilation rate throughout the treatment period. These results are consistent with previous reports showing a protective effect of estrogen against MFS associated aortic aneurysm progression (Saddic et al., 2023). Notably, the aortic dilation trajectory of 17β-estradiol-treated male mice closely resembled that of sham female mice, with comparable baseline-adjusted ATA dilation rates observed between the two groups. Furthermore, when diastolic diameter was normalized to body weight, the ratio between sham female and 17β-estradiol-treated male mice remained close to one at all examined ages, indicating that estrogen supplementation attenuated ATA dilation and restored aortic dilation rate toward the physiological growth phenotype. In contrast, OVX resulted in greater ATA dilation and increased dilation rate. The baseline diameter-adjusted diastolic dilation rate of OVX female mice was found to be closer to that of placebo-treated male mice (OVX vs. placebo: 0.0253 vs. 0.0294 mm/day). These findings emphasize the protective role of estrogen in slowing TAA growth and progression. Furthermore, the body weight-normalized diastolic diameter ratio between OVX female and placebo-treated male mice increased from 1.14 at 30 days to 1.35 at 60 days of age. These findings suggest that estrogen depletion can have a more pronounced effect on aneurysm progression than the baseline low-estrogen state of male mice, and that this detrimental effect becomes increasingly evident with age. This observation is consistent with clinical studies showing that the cardiovascular protection observed in premenopausal women diminishes after menopause, resulting in a substantial increase in aortic and cardiovascular disease risk (Stefanadis et al., 1999; Mehta et al., 2019). Lastly, the systolic dilation rate in female mice showed an interesting trend, with no significant differences observed between sham and OVX females. Together with the more pronounced differences in diastolic dilation rate, this finding suggests that estrogen depletion in females may predominantly influence the baseline geometry and passive structural remodeling of the aortic wall, accompanied by progressive wall stiffening, as observed in OVX female mice.

The progressive increase in ATA dilation observed in placebo-treated male and OVX female mice was consistent with significant increases in elastic fiber fragmentation identified histologically. As elastic fibers are the principal structural components responsible for aortic recoil and compliance (Yanagisawa and Wagenseil, 2020), their degradation impairs the capacity of the aortic wall to recover from repetitive pulsatile loading, leading to cumulative permanent deformation and progressive aneurysm enlargement with age. In contrast, 17β-estradiol-treated male mice and sham female mice maintained intact elastic fiber architecture and exhibited little or no age-related increase in ATA diameter, supporting a protective role of estrogen in preserving aortic structural integrity and limiting TAA progression.

The geometric remodeling of the ATA, quantified as the diastolic radius-to-thickness ratio, is a major determinant of circumferential wall stress according to the Laplace relationship (wall stress = blood pressure × radius/wall thickness). Because blood pressure was assumed to remain unchanged in this mouse model (Dwivedi et al., 2024), changes in the radius-to-thickness ratio directly reflect alterations in geometric remodeling caused by and/or leading to altered wall stresses. The radius-to-thickness ratio remained stable in 17β-estradiol-treated male mice and sham-operated female mice but increased progressively with age in placebo-treated male and OVX female mice, indicating that estrogen maintains aortic mechanical homeostasis by preserving the balance between vessel radius and wall thickness (Alford et al., 2007). Maintenance of this favorable geometry likely preserves physiological circumferential wall stress and supports normal vascular remodeling, as evidenced by intact elastic fiber architecture, preserved α-SMA amounts, and greater cellularity, all of which are indicative of maintenance of the vSMC contractile phenotype and ECM integrity. In contrast, estrogen deficiency resulted in adverse geometric remodeling, increased mechanical loading, elastic fiber fragmentation, decreased α-SMA amounts, and reduced cellularity, collectively compromising the mechanical and cellular integrity of the aortic wall and promoting progressive aneurysm enlargement. These structural and cellular alterations likely increase susceptibility to aortic rupture, ultimately contributing to the reduced survival observed in placebo-treated male and OVX female mice.

### 4.4 Estrogen preserves the ATA structural and material stiffness

The *in vivo* structural and material stiffness of the ATA wall increased progressively with age in placebo-treated male and OVX female mice, whereas these biomechanical parameters remained relatively unchanged in 17β-estradiol-treated male and sham female mice. These findings indicate that estrogen plays a critical role in maintaining aortic wall stiffness during aneurysm progression. The aorta functions as an elastic reservoir that stores part of the stroke volume during systole and releases it during diastole to maintain continuous blood flow throughout the cardiac cycle. Therefore, preservation of aortic stiffness that allows sufficient deformation is essential for normal vascular function, and our previous work demonstrated that lower aortic stiffness was associated with improved survival in MFS mice (Dwivedi et al., 2024).

Interestingly, the relative contributions of age and treatment differed between geometric and biomechanical outcomes for the ATA. For diastolic and systolic diameters, age showed the largest F-values in both male and female mice, indicating that aortic dilation was primarily driven by progressive disease advancement over time. In contrast, treatment exerted a substantially greater influence on structural and material stiffness, particularly in male mice, suggesting that estrogen modulation had a stronger effect on aortic wall stiffness than on geometry alone. Furthermore, the magnitude of the estrogen-mediated response differed between geometric and stiffness metrics. At 60 days of age, 17β-estradiol treatment reduced diastolic aortic diameter by approximately 1.3-fold relative to placebo-treated male mice but decreased incremental Young’s modulus by nearly 3-fold. Similarly, OVX female mice exhibited an approximately 1.8-fold greater diastolic diameter than sham-operated mice, whereas incremental Young’s modulus increased by nearly 5-fold. These findings demonstrate that alterations in estrogen levels produce substantially larger changes in aortic wall stiffness than in aortic diameter, suggesting that estrogen primarily preserves the functional and structural integrity of the aneurysmal wall rather than merely limiting aneurysm enlargement. This interpretation is further supported by the preservation of elastic fiber architecture, α-SMA protein expression, and cellularity in 17β-estradiol-treated male and sham-operated female mice, indicating that maintenance of vascular mechanical homeostasis may be a principal mechanism through which estrogen delays thoracic aortic aneurysm progression and improves survival.

Increased aortic stiffness not only reflects structural remodeling but also directly alters vascular hemodynamics. A stiffer aortic wall increases pulse wave velocity, resulting in earlier wave reflections and elevated circumferential wall stress, thereby increasing the mechanical burden experienced by the aneurysmal wall (Dwivedi et al., 2026). The progressive wall stiffening may be accompanied by increasingly disturbed flow patterns and reduced hemodynamic efficiency as we observed in our previous study (Dwivedi et al., 2026). Disturbed hemodynamics are well recognized to promote endothelial dysfunction and abnormal mechanotransduction (Chiu and Chien, 2011; Hsieh et al., 2014; Ando and Yamamoto, 2022), and may lead to altered endothelial and vSMC phenotypes and dysregulated ECM remodeling. Such maladaptive remodeling is characterized by elastic fiber fragmentation, medial collagen accumulation, loss of α-SMA protein expression, and reduced cellularity, all of which were observed in placebo-treated male and OVX female mice in the present study. These structural alterations may further increase wall stiffness, creating a positive feedback loop in which impaired wall mechanics promote disturbed hemodynamics, which in turn accelerate ECM degradation and vSMC phenotypic modulation. In contrast, estrogen supplementation was associated with preserved physiological wall mechanics leading to a more favorable hemodynamic environment, accompanied by intact elastic fiber architecture, higher α-SMA protein expression, and greater cellularity compared with placebo-treated male and OVX female mice.

Together with our previous observations (Dwivedi et al., 2026), these findings support a mechanistic model in which estrogen promotes vascular mechanical homeostasis, limits disturbed flow, and favors physiological ECM remodeling, thereby slowing ATA dilation, delaying aortic rupture, and improving survival in MFS mice. Conversely, estrogen deficiency was associated with progressive deterioration of wall mechanics, accompanied by elastic fiber fragmentation, reduced α-SMA expression, and decreased cellularity, ultimately contributing to age-dependent acceleration of thoracic aortic aneurysm progression and adverse outcomes.

The protective effects of estrogen on ECM remodeling, vSMC phenotype modulation, aortic wall mechanics, dilation, and rupture may, at least in part, be mediated through suppression of inflammatory signaling (Saddic et al., 2023; Sazonova et al., 2024). Previous studies have demonstrated a close association between inflammatory activity, ECM remodeling, and altered biomechanical properties of the ascending aortic wall (Karas et al., 2001). Moreover, increased expression of inflammatory and remodeling-related genes has been reported following OVX, whereas estrogen replacement attenuated these responses (Hamilton et al., 2008). Importantly, in the *Fbn1^C1041G/+^* Marfan mouse model 17β-estradiol treatment reduced aortic root dilation and rupture while suppressing TNF-α/NF-κB-mediated inflammatory signaling and inflammatory vSMC phenotypic modulation (Saddic et al., 2023). OVX has also been associated with increased arterial stiffness, further suggesting that ovarian hormone depletion may influence both vascular inflammatory remodeling and mechanical properties (Kilanowski-Doroh et al., 2024). A recent study in a chemical mouse model of aortic aneurysm and dissection further supports a protective role of estrogen in aortic disease, demonstrating that 17β-estradiol treatment in OVX female mice attenuated thoracic aortic dilation and was associated with preservation of the contractile vSMC phenotype and reduced phenotypic switching (Pu et al., 2025). These findings are consistent with our observations and suggest that estrogen-mediated regulation of vSMC phenotype and associated vascular remodeling may contribute to preservation of aortic wall structure and mechanics.

The present study does not isolate the effects of depletion of other ovarian hormones following OVX, which may also contribute to alterations in aortic dilation and wall biomechanics through associated inflammatory pathways (Karas et al., 2001; Sazonova et al., 2024). OVX can produce a broad ovarian hormone-depletion state rather than selective estrogen deficiency. Previous studies have demonstrated a role for progesterone receptor signaling in vascular remodeling in OVX mice by changing the vSMC proliferation and response to vascular injury (Karas et al., 2001). In addition, OVX increased arterial material stiffness and downregulated vSMC contractile genes, while pharmacological activation of estrogen signaling through GPER reversed arterial stiffening and restored contractile gene expression (Kilanowski-Doroh et al., 2024). There is evidence that estrogen replacement following OVX can attenuate adverse cellular and molecular remodeling, including inflammatory gene expression and pathological vSMC phenotypic switching. Estrogen preserved the contractile vSMC phenotype through ERα/CREB signaling and attenuated aortic pathological remodeling, while estrogen replacement following OVX suppressed inflammation-related cardiac gene expression (Pu et al., 2025). These findings support a potential protective role of estrogen in regulating cellular phenotype and inflammatory remodeling following ovarian hormone depletion. The direct effect of ovarian hormones other than estrogen toward aortic ECM remodeling, aneurysm progression and outcome is not well-understood and future studies incorporating selective replacement or manipulation of individual ovarian hormones, particularly estrogen and progesterone, will be important to distinguish their respective contributions to ATA remodeling and biomechanics.

In addition to sex hormones, hormone-independent mechanisms, particularly sex chromosome complement, may contribute to the observed sexual dimorphism in TAA outcomes. Recent studies using Four Core Genotype mice demonstrated that XX versus XY chromosome complement influences thoracic aortic stiffness and ECM-related gene expression, suggesting that sex chromosomes can independently modulate aortic structural and mechanical properties (Kamau et al., 2025). Similarly, Alsiraj et al. (2017). demonstrated that phenotypic female mice with an XY chromosome complement develop more severe experimental abdominal aortic aneurysms than XX females, accompanied by differences in inflammatory signaling, matrix metalloproteinase activity, and aortic gene expression. Furthermore, incomplete X-chromosome inactivation and the resulting increased dosage of X-linked escape genes have emerged as potential contributors to cardiovascular sexual dimorphism (Spiering et al., 2026). Collectively, these findings suggest that sex chromosome complement and X-linked gene dosage, independently or in interaction with gonadal hormones, may influence aortic remodeling and contribute to sex differences in MFS-associated TAA. Further studies are warranted to explain the relative contributions and interactions of sex chromosomes and sex hormones in TAA development and progression.

## 5 Limitations

This study has several limitations. First, the statistical power decreased with age because of progressive mortality, particularly in placebo-treated male mice; therefore, quantitative analyses were limited to 60 days of age. Second, diastolic wall thickness was measured from ultrasound images, and the relatively low spatial resolution may introduce errors in the calculation of incremental Young’s modulus. However, the values obtained in the present study were comparable to those reported in our previous ex vivo mechanical experiments, where wall thickness was measured directly (Dwivedi et al., 2024; Dwivedi et al., 2025). Third, pulse pressure was not measured individually for each mouse but was assigned the average age- and sex-specific value obtained from our previous study with *Fbn1^mgR/mgR^* mice (Dwivedi et al., 2024), which will influence the estimated stiffness parameters, Peterson’s modulus and incremental Young’s modulus. This limitation is particularly relevant because ovarian hormone status can influence systemic blood pressure. Previous mouse studies reported increased blood pressure following OVX; for example, systolic blood pressure increased from approximately 107 to 120 mmHg following OVX in wild-type female mice and was restored to approximately 104 mmHg with estrogen replacement (Athirakul et al., 2008). Similarly, OVX increased mean arterial pressure in a mouse model of hypertension, whereas 17β-estradiol replacement prevented this increase (Gilbert et al., 2014). However, these effects are model dependent, as other studies have reported OVX-induced arterial stiffening without significant changes in blood pressure (Kilanowski-Doroh et al., 2024). Therefore, potential treatment-related differences in pulse pressure in the present study cannot be excluded and may have introduced bias into the calculations of the pressure-dependent stiffness parameters. Although the observed treatment-related trends in aortic stiffness are supported by the accompanying changes in aortic geometry and wall remodeling, individually measured blood pressures are required to determine the precise magnitude of changes in Peterson’s modulus and incremental Young’s modulus as a result of the applied treatments. Fourth, constant-release 17β-estradiol delivery does not fully reproduce the physiological temporal fluctuations in estrogen levels, while OVX induces an abrupt loss of ovarian hormones rather than the gradual hormonal decline associated with human reproductive aging and menopause. These inherent features of the experimental models should be considered when interpreting the findings in the context of human hormonal physiology and their translational relevance. Finally, this study includes tissue-level structural and biomechanical observations and does not elucidate the molecular mechanisms underlying the protective effects of estrogen. Future studies are needed to investigate the molecular mechanisms of estrogen-mediated protection in both male and female mice, and particularly the roles of mechanobiology and mechanotransduction pathways.

## 6 Conclusion

This study investigated the role of estrogen in TAA development and progression through 17β-estradiol supplementation in male and estrogen depletion by bilateral OVX in female *Fbn1^mgR/mgR^* mice. Our findings demonstrate a significant protective effect of increased estrogen on ECM remodeling, particularly elastic fiber integrity, α-SMA amounts indicating contractile vSMC phenotype, and aortic wall stiffness, resulting in slower ATA dilation and prolonged survival in 17β-estradiol-treated male mice compared with placebo mice. In contrast, estrogen depletion accelerated elastic fiber fragmentation, reduced α-SMA amounts, increased aortic wall stiffness, promoted severe ATA dilation, and shortened survival in OVX female mice compared with sham-operated female mice. Overall, this study demonstrates the protective role of estrogen in preserving ATA wall stiffness, ECM integrity, and vascular cellularity, thereby attenuating TAA progression and improving survival.

## Data Availability

The original contributions presented in the study are included in the article/**Supplementary Material**. Further inquiries can be directed to the corresponding author.

## References

[B1] Alford P. W. Humphrey J. D. Taber L. A. (2007). Growth and remodeling in a thick-walled artery model: Effects of spatial variations in wall constituents. Biomech. Model. Mechanobiol. doi: 10.1007/s10237-007-0101-2 17786493 PMC2594015

[B2] Alsiraj Y. Thatcher S. E. Charnigo R. Chen K. Blalock E. Daugherty A. . (2017). Female mice with an XY sex chromosome complement develop severe angiotensin II-induced abdominal aortic aneurysms. Circulation 135, 379–391. doi: 10.1161/circulationaha.116.023789 27815372 PMC5470072

[B3] Ando J. Yamamoto K. (2022). Hemodynamic forces, endothelial mechanotransduction, and vascular diseases. Magn. Reson. Med. Sci. 21, 258–266. doi: 10.2463/mrms.rev.2021-0018 34024868 PMC9680547

[B4] Athirakul K. Bradbury J. A. Graves J. P. Degraff L. M. Ma J. Zhao Y. . (2008). Increased blood pressure in mice lacking cytochrome P450 2J5. FASEB J. 22, 4096–4108. doi: 10.1096/fj.08-114413 18716027 PMC2614614

[B5] Boese A. C. Kim S. C. Yin K. J. Lee J. P. Hamblin M. H. (2017). Sex differences in vascular physiology and pathophysiology: Estrogen and androgen signaling in health and disease. Am. J. Physiol. Heart Circ. Physiol. 313, H524–H545. doi: 10.1152/ajpheart.00217.2016 28626075 PMC5625178

[B6] Bollache E. Guzzardi D. G. Sattari S. Olsen K. E. Di Martino E. S. Malaisrie S. C. . (2018). Aortic valve-mediated wall shear stress is heterogeneous and predicts regional aortic elastic fiber thinning in bicuspid aortic valve-associated aortopathy. J. Thorac. Cardiovasc. Surg. 156, 2112–2120. doi: 10.1016/j.jtcvs.2018.05.095 30060930 PMC6242768

[B7] Chiu J. J. Chien S. (2011). Effects of disturbed flow on vascular endothelium: Pathophysiological basis and clinical perspectives. Physiol. Rev. 91, 327–387. doi: 10.1152/physrev.00047.2009 21248169 PMC3844671

[B8] Chung J. Coutinho T. Chu M. W.A. Ouzounian M. (2020). Sex differences in thoracic aortic disease: A review of the literature and a call to action. J. Thorac. Cardiovasc. Surg. 160, 656–660. doi: 10.1016/j.jtcvs.2019.09.194 31987620

[B9] Crosier R. Lopez Laporte M. A. Unni R. R. Coutinho T. (2024). Female-specific considerations in aortic health and disease. CJC Open 6, 391–406. doi: 10.1016/j.cjco.2023.09.006 38487044 PMC10935703

[B10] Crousillat D. Briller J. Aggarwal N. Cho L. Coutinho T. Harrington C. . (2023). Sex differences in thoracic aortic disease and dissection: JACC review topic of the week. J. Am. Coll. Cardiol. 82, 817–827. doi: 10.1016/j.jacc.2023.05.067 37612014

[B11] Detaint D. Faivre L. Collod-Beroud G. Child A. H. Loeys B. L. Binquet C. . (2010). Cardiovascular manifestations in men and women carrying a FBN1 mutation. Eur. Heart J. 31, 2223–2229. doi: 10.1093/eurheartj/ehq258 20709720

[B12] Dwivedi K. K. Rother J. Wagenseil J. E. (2024). Age- and sex-specific biomechanics and extracellular matrix remodeling of the ascending aorta in a mouse model of severe Marfan syndrome. Am. J. Physiol. Heart Circ. Physiol. 327, H1037–H1051. doi: 10.1152/ajpheart.00391.2024 39212766 PMC11482245

[B13] Dwivedi K. K. Rother J. Wagenseil J. E. (2025). Sex- and region-specific differences in microstructural remodeling and passive biomechanics of the aorta correlate with aneurysm propensity in a mouse model of severe Marfan syndrome. Acta Biomater. 200, 218–235. doi: 10.1016/j.actbio.2025.05.056 40414262 PMC12416491

[B14] Dwivedi K. K. Wu Y. Bazzi M. S. Wiputra H. Rother J. Quirk J. D. . (2026). Alterations in ascending aortic hemodynamics and aortic length correlate with sex-specific thoracic aortic aneurysm dilation and lifespan in a mouse model of severe Marfan syndrome. Comput. Biol. Med. 205, 111594. doi: 10.1016/j.compbiomed.2026.111594 41775185 PMC13081028

[B15] Gharraee N. Sun Y. Swisher J. A. Lessner S. M. (2022). Age and sex dependency of thoracic aortopathy in a mouse model of Marfan syndrome. Am. J. Physiol. Heart Circ. Physiol. 322, H44–H56. doi: 10.1152/ajpheart.00255.2021 34714692 PMC8698500

[B16] Gilbert E. L. Mathis K. W. Ryan M. J. (2014). 17beta-estradiol protects against the progression of hypertension during adulthood in a mouse model of systemic lupus erythematosus. Hypertension 63, 616–623. doi: 10.1161/hypertensionaha.113.02385 24366082 PMC4365871

[B17] Gonzales R. J. Walker B. R. Kanagy N. L. (2001). 17beta-estradiol increases nitric oxide-dependent dilation in rat pulmonary arteries and thoracic aorta. Am. J. Physiol. Lung Cell. Mol. Physiol. 280, L555–L564. doi: 10.1152/ajplung.2001.280.3.l555 11159040

[B18] Gosling R. G. Budge M. M. (2003). Terminology for describing the elastic behavior of arteries. Hypertension 41, 1180–1182. doi: 10.1161/01.hyp.0000072271.36866.2a 12756217

[B19] Gouveia E. M.R. Silva Duarte G. Lopes A. Alves M. Caldeira D. Fernandes E. F.R. . (2022). Incidence and prevalence of thoracic aortic aneurysms: A systematic review and meta-analysis of population-based studies. Semin. Thorac. Cardiovasc. Surg. 34, 1–16. doi: 10.1053/j.semtcvs.2021.02.029 33705940

[B20] Hamilton K. L. Lin L. Wang Y. Knowlton A. A. (2008). Effect of ovariectomy on cardiac gene expression: Inflammation and changes in SOCS gene expression. Physiol. Genomics 32, 254–263. doi: 10.1152/physiolgenomics.00039.2007 17986523

[B21] Hsieh H. J. Liu C. A. Huang B. Tseng A. H. Wang D. L. (2014). Shear-induced endothelial mechanotransduction: The interplay between reactive oxygen species (ROS) and nitric oxide (NO) and the pathophysiological implications. J. Biomed. Sci. 21, 3. doi: 10.1186/1423-0127-21-3 24410814 PMC3898375

[B22] Huang W. Zhao S. Liu H. Pan M. Dong H. (2024). The role of protein degradation in estimation postmortem interval and confirmation of cause of death in forensic pathology: A literature review. Int. J. Mol. Sci. 25. doi: 10.3390/ijms25031659 38338938 PMC10855206

[B23] Jimenez-Altayo F. Siegert A. M. Bonorino F. Meirelles T. Barbera L. Dantas A. P. . (2017). Differences in the thoracic aorta by region and sex in a murine model of Marfan syndrome. Front. Physiol. 8, 933. doi: 10.3389/fphys.2017.00933 PMC5694786 29187826

[B24] Kamau A. N. Sakamuri A. Okoye D. O. Sengottaian D. Cannon J. Guerrero-Millan J. . (2025). Sexual dimorphism in the downregulation of extracellular matrix genes contributes to aortic stiffness in female mice. Am. J. Physiol. Heart Circ. Physiol. 328, H472–H483. doi: 10.1152/ajpheart.00432.2024 39873616 PMC12276896

[B25] Karas R. H. Van Eickels M. Lydon J. P. Roddy S. Kwoun M. Aronovitz M. . (2001). A complex role for the progesterone receptor in the response to vascular injury. J. Clin. Invest. 108, 611–618. doi: 10.1172/jci200111374 11518735 PMC209395

[B26] Kilanowski-Doroh I. M. Mcnally A. B. Wong T. J. Visniauskas B. Blessinger S. A. Imulinde Sugi A. . (2024). Ovariectomy-induced arterial stiffening differs from vascular aging and is reversed by GPER activation. Hypertension 81, e51–e62. doi: 10.1161/hypertensionaha.123.22024 38445498 PMC11023783

[B27] Laser A. Ghosh A. Roelofs K. Sadiq O. Mcevoy B. Dimusto P. . (2014). Increased estrogen receptor alpha in experimental aortic aneurysms in females compared with males. J. Surg. Res. 186, 467–474. doi: 10.1016/j.jss.2013.07.050 23993200 PMC3858525

[B28] Martin-McNulty B. Tham D. M. Da Cunha V. Ho J. J. Wilson D. W. Rutledge J. C. . (2003). 17 beta-estradiol attenuates development of angiotensin II-induced aortic abdominal aneurysm in apolipoprotein E-deficient mice. Arterioscler. Thromb. Vasc. Biol. 23, 1627–1632. doi: 10.1161/01.atv.0000085842.20866.6a 12855485

[B29] Mehta J. M. Chester R. C. Kling J. M. (2019). The timing hypothesis: Hormone therapy for treating symptomatic women during menopause and its relationship to cardiovascular disease. J. Womens Health (Larchmt) 28, 705–711. doi: 10.1089/jwh.2018.7201 30484736

[B30] Milewicz D. M. Guo D. C. Tran-Fadulu V. Lafont A. L. Papke C. L. Inamoto S. . (2008). Genetic basis of thoracic aortic aneurysms and dissections: Focus on smooth muscle cell contractile dysfunction. Annu. Rev. Genomics Hum. Genet. 9, 283–302. doi: 10.1146/annurev.genom.8.080706.092303 18544034

[B31] Natoli A. K. Medley T. L. Ahimastos A. A. Drew B. G. Thearle D. J. Dilley R. J. . (2005). Sex steroids modulate human aortic smooth muscle cell matrix protein deposition and matrix metalloproteinase expression. Hypertension 46, 1129–1134. doi: 10.1161/01.hyp.0000187016.06549.96 16230520

[B32] Nienaber C. A. Fattori R. Mehta R. H. Richartz B. M. Evangelista A. Petzsch M. . (2004). Gender-related differences in acute aortic dissection. Circulation 109, 3014–3021. doi: 10.1016/j.accreview.2004.08.086 15197151

[B33] Pereira L. Lee S. Y. Gayraud B. Andrikopoulos K. Shapiro S. D. Bunton T. . Pathogenetic sequence for aneurysm revealed in mice underexpressing fibrillin-1. Proc Natl Acad Sci U S A 96, 3819–3823. doi: 10.1073/pnas.96.7.3819 PMC22378 10097121

[B34] Pereira L. Andrikopoulos K. Tian J. Lee S. Y. Keene D. R. Ono R. . (1997). Targetting of the gene encoding fibrillin-1 recapitulates the vascular aspect of Marfan syndrome. Nat. Genet. 17, 218–222. doi: 10.1038/ng1097-218 9326947

[B35] Pu Y. Zhou Y. Guo T. Chai X. Yang G. (2025). Estrogen inhibits the phenotypic switching of vascular smooth muscle cells through ER-alpha/CREB in aortic dissection. ACS Omega 10, 15256–15271. doi: 10.1021/acsomega.4c10955 40290976 PMC12019514

[B36] Qi X. Wang F. Chun C. Saldarriaga L. Jiang Z. Pruitt E. Y. . (2020). A validated mouse model capable of recapitulating the protective effects of female sex hormones on ascending aortic aneurysms and dissections (AADs). Physiol. Rep. 8, e14631. doi: 10.14814/phy2.14631 33242364 PMC7690909

[B37] Ramirez F. Dietz H. C. (2007). Marfan syndrome: From molecular pathogenesis to clinical treatment. Curr. Opin. Genet. Dev. 17, 252–258. doi: 10.1016/j.gde.2007.04.006 17467262

[B38] Rodriguez-Landa J. F. (2022). Considerations of timing post-ovariectomy in mice and rats in studying anxiety- and depression-like behaviors associated with surgical menopause in women. Front. Behav. Neurosci. 16, 829274. doi: 10.3389/fnbeh.2022.829274 35309685 PMC8931748

[B39] Roman M. J. Devereux R. B. Preiss L. R. Asch F. M. Eagle K. A. Holmes K. W. . (2017). Associations of age and sex with Marfan phenotype: The National Heart, Lung, and Blood Institute GenTAC (Genetically Triggered Thoracic Aortic Aneurysms and Cardiovascular Conditions) Registry. Circ. Cardiovasc. Genet. 10. 10.1161/CIRCGENETICS.116.001647 PMC5500868 28600386

[B40] Saddic L. Escopete S. Zilberberg L. Kalsow S. Gupta D. Eghbali M. . (2023). 17 beta-estradiol impedes aortic root dilation and rupture in male Marfan mice. Int. J. Mol. Sci. 24. doi: 10.1152/physiol.2023.38.s1.5735279 PMC10487461 37686377

[B41] Sazonova S. I. Saushkin V. V. Panfilov D. S. Stepanov I. V. Gusakova A. M. Skosyrsky A. B. . (2024). Insights into ascending aortic aneurysm: Interactions between biomechanical properties of the aortic wall and tissue biomarkers. Heliyon 10, e23538. doi: 10.1016/j.heliyon.2023.e23538 38170063 PMC10758872

[B42] Seeburun S. Wu S. Hemani D. Pham L. Ju D. Xie Y. . (2023). Insights into elastic fiber fragmentation: Mechanisms and treatment of aortic aneurysm in Marfan syndrome. Vascul Pharmacol. 153, 107215. doi: 10.1016/j.vph.2023.107215 37640090 PMC10872825

[B43] Spiering A. E. Groenheide P. J. Mokry M. Onland-Moret N. C. Civelek M. Reue K. . (2026). Sex chromosomes and cardiovascular disease. Eur. J. Prev. Cardiol. 33, 316–326. doi: 10.1093/eurjpc/zwaf224 40231569

[B44] Stefanadis C. Tsiamis E. Dernellis J. Toutouzas P. (1999). Effect of estrogen on aortic function in postmenopausal women. Am. J. Physiol. 276, H658–H662. doi: 10.1152/ajpheart.1999.276.2.h658 9950868

[B45] Weiss D. Rego B. V. Cavinato C. Li D. S. Kawamura Y. Emuna N. . (2023). Effects of age, sex, and extracellular matrix integrity on aortic dilatation and rupture in a mouse model of Marfan syndrome. Arterioscler. Thromb. Vasc. Biol. 43, e358–e372. doi: 10.1161/atvbaha.123.319122 37470181 PMC10528515

[B46] White S. E. Karbasion N. Snider J. C. Florian-Rodriguez M. Bersi M. R. Miller K. S. (2024). Remodeling of murine vaginal smooth muscle function with reproductive age and elastic fiber disruption. Acta Biomater. 175, 186–198. doi: 10.1016/j.actbio.2023.12.035 38151068

[B47] Xing D. Nozell S. Chen Y. F. Hage F. Oparil S. (2009). Estrogen and mechanisms of vascular protection. Arterioscler. Thromb. Vasc. Biol. 29, 289–295. doi: 10.1161/atvbaha.108.182279 19221203 PMC2700771

[B48] Yanagisawa H. Wagenseil J. (2020). Elastic fibers and biomechanics of the aorta: Insights from mouse studies. Matrix Biol. 85-86, 160–172. doi: 10.1016/j.matbio.2019.03.001 30880160 PMC6745291

