# Supplementary material for: Estrogen attenuates thoracic aortic aneurysm progression through reduced extracellular matrix remodeling and wall stiffening in a severe Marfan syndrome mouse model
Source: Front Physiol. 2026 Oct 30;17:1944714. doi: 10.3389/fphys.2026.1944714 (PMC13759295; doi:10.3389/fphys.2026.1944714)
Supplement: Supplementary file 1 [file DataSheet1.pdf]

Supplementary Data

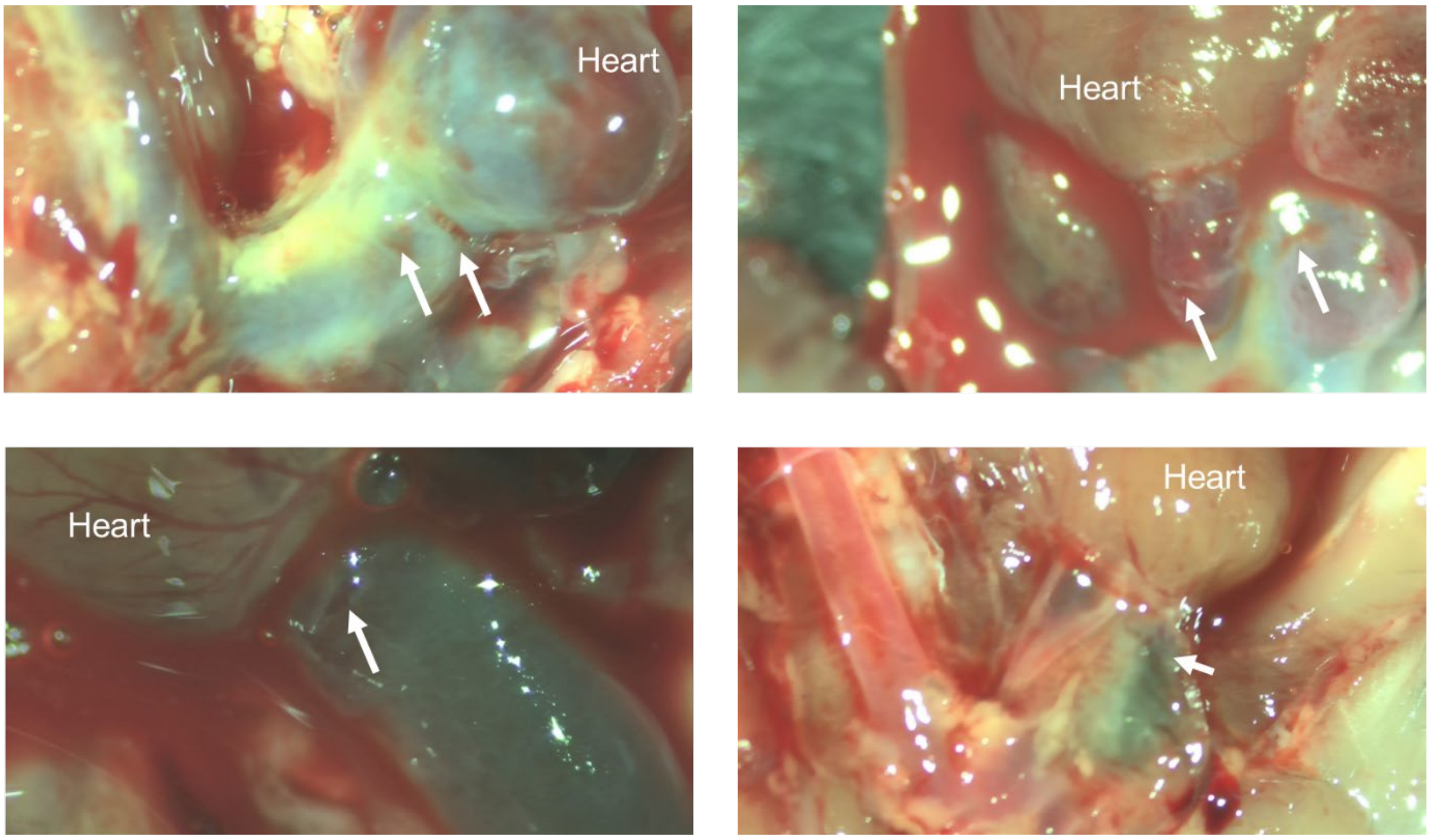

Supplementary Figure S1. Representative images of the ascending thoracic aorta (ATA) demonstrating the site of aortic rupture, indicated by the white arrow

Table S1. Individual ascending thoracic aortic diastolic diameters in placebo male mice. ATA diastolic diameter (mm) was measured longitudinally at 30, 45, 60, 75, and 90 days of age. Values are presented for individual mice, with group data summarized as mean ± SD at each time point. A missing value at a subsequent time point indicates that the animal died after its last available measurement and before the next scheduled measurement.

Diastolic diameter (mm)	Age (days)				
**Mouse ID**	**30**	**45**	**60**	**75**	**90**
39031-1M	1.34	1.72			
39035-4M	1.40	1.41			
39080-1M	1.55	2.72	3.31		
39086-2M	1.40	1.53	2.04	2.18	2.37
39104-1M	1.51	2.00			
39150-2M	1.35	1.40	1.44	2.15	2.25
39150_4M	1.23	1.51	1.92	2.07	2.15
39152-1M	2.01	3.01			
39160-2M	1.57	2.07	3.14		
39182_3M	1.44	1.78	1.94	2.01	
39192-1M	1.52	2.41	2.88		
**Mean**	**1.48**	**1.96**	**2.38**	**2.10**	**2.26**
**SD**	**0.19**	**0.52**	**0.62**	**0.06**	**0.08**

Table S2. Individual ascending thoracic aortic diastolic diameters in 17β-Estradiol male mice. ATA diastolic diameter (mm) was measured longitudinally at 30, 45, 60, 75, and 90 days of age. Values are presented for individual mice, with group data summarized as mean ± SD at each time point. A missing value at a subsequent time point indicates that the animal died after its last available measurement and before the next scheduled measurement.

**Diastolic diameter (mm)**	**Age (days)**				
**Mouse ID**	**30**	**45**	**60**	**75**	**90**
39034-2M	1.33	1.48	1.51	1.54	1.56
39041-1M	1.40	1.41	1.47	1.48	2.07
39047-3M	1.20	1.53	1.56	1.83	1.98
39058-1M	1.44	1.50	1.83	1.90	2.00
39084-3M	1.32	1.35	1.38	1.48	1.49
39102-4M	1.63	2.11	2.25	2.25	
39106-1M	1.68	2.16	2.79	3.56	
39126-1M	1.54	1.56	1.59	1.61	1.60
39138-1M	1.59	1.83	2.24	2.49	2.59
39148_2M	1.49	1.64	2.18	2.21	
39162-2M	1.36	1.45	1.64	1.68	1.76
39192A_2M	1.40	1.56	1.60	1.68	1.82
**Mean**	**1.45**	**1.63**	**1.84**	**1.98**	**1.87**
**SD**	**0.14**	**0.25**	**0.41**	**0.57**	**0.32**

Table S3. Individual ascending thoracic aortic diastolic diameters in sham female mice. ATA diastolic diameter (mm) was measured longitudinally at 30, 45, 60, 75, and 90 days of age. Values are presented for individual mice, with group data summarized as mean ± SD at each time point.

**Diastolic diameter (mm)**	**Age (days)**				
**Mouse ID**	**30**	**45**	**60**	**75**	**90**
39042-3F	1.29	1.30	1.37	1.43	1.54
39101-2F	1.46	1.73	1.78	1.81	1.95
39083-3F	1.18	1.37	1.61	1.64	1.73
39117-1F	1.45	1.81	1.93	1.96	2.34
39147-1F	1.34	1.33	1.34	1.35	1.39
39161-2F	1.33	1.50	1.56	1.64	1.78
39171_3F	1.38	1.47	1.51	1.74	2.05
**Mean**	**1.35**	**1.50**	**1.59**	**1.65**	**1.83**
**SD**	**0.09**	**0.18**	**0.20**	**0.20**	**0.30**

Table S4. Individual ascending thoracic aortic diastolic diameters in OVX female mice. ATA diastolic diameter (mm) was measured longitudinally at 30, 45, 60, 75, and 90 days of age. Values are presented for individual mice, with group data summarized as mean ± SD at each time point. A missing value at a subsequent time point indicates that the animal died after its last available measurement and before the next scheduled measurement.

**Diastolic diameter (mm)**	**Age (days)**				
**Mouse ID**	**30**	**45**	**60**	**75**	**90**
39030-2F	1.29	1.94	2.74	2.91	
39053-1F	1.38	1.54	1.91	2.18	2.39
39060-2F	1.53	1.87	2.27	2.53	2.76
39060-5F	1.52	1.67	2.07	2.23	2.35
39085-3F	1.60	1.76	2.08	2.39	2.57
39115-1F	1.85	2.44			
39125_1F	1.80	3.43	3.81	4.16	4.27
39149_1F	1.28	1.45	2.07	2.27	2.43
39169_1F	1.63	2.10			
39161_1F	1.27	1.72			
39187_1F	1.62	2.11	2.48	2.68	2.88
**Mean**	**1.52**	**2.00**	**2.43**	**2.67**	**2.81**
**SD**	**0.19**	**0.53**	**0.58**	**0.61**	**0.62**

Table S5. Individual ascending thoracic aortic systolic diameters in placebo male mice. ATA diastolic diameter (mm) was measured longitudinally at 30, 45, 60, 75, and 90 days of age. Values are presented for individual mice, with group data summarized as mean ± SD at each time point. A missing value at a subsequent time point indicates that the animal died after its last available measurement and before the next scheduled measurement

**Systolic diameter (mm)**	**Age (days)**				
**Mouse ID**	**30**	**45**	**60**	**75**	**90**
39031-1M	1.53	1.83			
39035-4M	1.46	2.28			
39080-1M	1.71	2.85	3.40		
39086-2M	1.58	2.10	2.23	2.42	2.40
39104-1M	1.69	2.07			
39150-2M	1.47	1.51	1.65	2.30	2.35
39150-4M	1.36	2.05	2.25	2.35	2.45
39152-1M	2.24	3.14			
39160-2M	1.71	2.16	3.26		
39182_3M	1.62	1.91	2.13	2.19	
39192-1M	1.69	2.53	3.01		
**Mean**	**1.64**	**2.22**	**2.56**	**2.32**	**2.40**
**SD**	**0.22**	**0.44**	**0.57**	**0.07**	**0.04**

Table S6. Individual ascending thoracic aortic systolic diameters in 17β-Estradiol male mice. ATA diastolic diameter (mm) was measured longitudinally at 30, 45, 60, 75, and 90 days of age. Values are presented for individual mice, with group data summarized as mean ± SD at each time point. A missing value at a subsequent time point indicates that the animal died after its last available measurement and before the next scheduled measurement.

**Systolic diameter (mm)**	**Age (days)**				
**Mouse ID**	**30**	**45**	**60**	**75**	**90**
39034-2M	1.47	1.59	1.88	1.93	1.98
39041-1M	1.56	1.52	1.61	1.68	2.15
39047-3M	1.33	1.65	1.85	1.99	2.11
39058-1M	1.60	1.62	1.99	2.01	2.13
39084-3M	1.45	1.46	1.52	1.68	1.96
39102-4M	1.78	2.24	2.43	2.35	
39106-1M	1.77	2.26	2.91	3.71	
39126-1M	1.71	1.77	1.85	1.92	2.05
39138-1M	1.75	2.01	2.41	2.66	2.76
39148_2M	1.75	1.86	2.33	2.24	
39162-2M	1.62	1.81	1.91	1.97	1.98
39192A_2M	1.63	1.74	1.81	1.89	2.01
**Mean**	**1.62**	**1.79**	**2.04**	**2.17**	**2.13**
**SD**	**0.14**	**0.25**	**0.38**	**0.53**	**0.23**

Table S7. Individual ascending thoracic aortic systolic diameters in sham female mice. ATA diastolic diameter (mm) was measured longitudinally at 30, 45, 60, 75, and 90 days of age. Values are presented for individual mice, with group data summarized as mean ± SD at each time point.

**Systolic diameter (mm)**	**Age (days)**				
**Mouse ID**	**30**	**45**	**60**	**75**	**90**
39042-3F	1.42	1.44	1.49	1.53	1.66
39101-2F	1.56	1.80	1.85	1.89	2.19
39083-3F	1.29	1.52	1.98	2.06	2.16
39117-1F	1.59	1.97	2.06	2.33	2.45
39147-1F	1.48	1.52	1.71	1.73	1.79
39161-2F	1.51	1.64	1.74	1.80	1.83
39171_3F	1.48	1.64	1.68	1.89	2.44
**Mean**	**1.48**	**1.65**	**1.79**	**1.89**	**2.07**
**SD**	**0.09**	**0.17**	**0.18**	**0.23**	**0.29**

Table S8. Individual ascending thoracic aortic systolic diameters in OVX female mice. ATA diastolic diameter (mm) was measured longitudinally at 30, 45, 60, 75, and 90 days of age. Values are presented for individual mice, with group data summarized as mean ± SD at each time point. A missing value at a subsequent time point indicates that the animal died after its last available measurement and before the next scheduled measurement.

**Systolic diameter (mm)**	**Age (days)**				
Mouse ID	30	45	60	75	90
39030-2F	1.56	2.05	2.88	2.98	
39053-1F	1.56	1.66	1.87	1.93	2.58
39060-2F	1.72	2.11	2.41	2.56	2.81
39060-5F	1.61	1.76	1.85	2.10	2.46
39085-3F	1.71	1.66	2.06	2.35	2.43
39115-1F	2.00	2.47			
39125_1F	1.97	3.50	3.87	4.21	4.32
39149_1F	1.46	1.65	1.81	1.94	2.41
39169_1F	1.80	2.13			
39161_1F	1.45	1.75			
39187_1F	1.75	2.26	2.54	2.65	2.73
**Mean**	**1.69**	**2.09**	**2.41**	**2.59**	**2.82**
**SD**	**0.18**	**0.52**	**0.66**	**0.70**	**0.63**

Table S9. Individual ascending thoracic aortic diastolic dilation rates for placebo male mice. Absolute dilation rate (mm)/day, percentage dilation rate (%)/day and adjusted (adj) dilation rate(mm/mm)/day are shown.

**Diastolic dilation rates**					
**Mouse ID**	**Absolute rate (mm/day)**	**Diam at 30 days**	**Percentage rate (%/day)**	**Adj baseline diam**	**Adj rate (mm/mm/day)**
39031-1M	0.025	1.340	1.891	1.461	0.035
39035-4M	0.011	1.400	0.762	1.461	0.011
39080-1M	0.059	1.550	3.785	1.461	0.051
39086-2M	0.017	1.400	1.233	1.461	0.022
39104-1M	0.033	1.510	2.163	1.461	0.029
39150-2M	0.017	1.350	1.259	1.461	0.026
39150-4M	0.016	1.230	1.301	1.461	0.035
39152-1M	0.067	2.010	3.317	1.461	0.021
39160-2M	0.052	1.570	3.333	1.461	0.043
39182_3M	0.012	1.440	0.866	1.461	0.014
39192-1M	0.045	1.520	2.982	1.461	0.040
**Mean**	**0.032**	**1.484**	**2.081**		**0.0294**
**SD**	**0.019**	**0.193**	**1.048**		**0.012**

Table S10. Individual ascending thoracic aortic diastolic dilation rates for 17β-Estradiol male mice. Absolute dilation rate (mm)/day, percentage dilation rate (%)/day and adjusted (adj) dilation rate(mm/mm)/day are shown.

Diastolic dilation rates					
**Mouse ID**	**Absolute rate (mm/day)**	**Diam at 30 days**	**Percentage rate (%/day)**	**Adj baseline diam**	**Adj rate (mm/mm/day)**
39034-2M	0.003	1.330	0.261	1.461	0.009
39041-1M	0.009	1.400	0.671	1.461	0.012
39047-3M	0.012	1.200	1.033	1.461	0.024
39058-1M	0.010	1.440	0.704	1.461	0.011
39084-3M	0.003	1.320	0.237	1.461	0.010
39102-4M	0.013	1.630	0.818	1.461	0.006
39106-1M	0.042	1.680	2.488	1.461	0.032
39126-1M	0.001	1.540	0.074	1.461	0.002
39138-1M	0.018	1.590	1.115	1.461	0.012
39148_2M	0.018	1.490	1.208	1.461	0.017
39162-2M	0.007	1.360	0.505	1.461	0.011
39192A_2M	0.006	1.400	0.457	1.461	0.009
**Mean**	**0.012**	**1.448**	**0.798**		**0.0126**
**SD**	**0.010**	**0.136**	**0.615**		**0.008**

Table S11. Individual ascending thoracic aortic diastolic dilation rates for sham female mice. Absolute dilation rate (mm)/day, percentage dilation rate (%)/day and adjusted (adj) dilation rate(mm/mm)/day are shown.

Diastolic dilation rates					
**Mouse ID**	**Absolute rate (mm/day)**	**Diam at 30 days**	**Percentage rate (%/day)**	**Adj baseline diam**	**Adj rate (mm/mm/day)**
39042-3F	0.004	1.290	0.326	1.461	0.006
39101-2F	0.009	1.460	0.626	1.461	0.012
39083-3F	0.007	1.180	0.599	1.461	0.007
39117-1F	0.013	1.450	0.887	1.461	0.013
39147-1F	0.001	1.340	0.060	1.461	0.002
39161-2F	0.007	1.330	0.521	1.461	0.008
39171_3F	0.011	1.380	0.778	1.461	0.012
**Mean**	**0.007**	**1.347**	**0.542**		**0.0086**
**SD**	**0.004**	**0.089**	**0.258**		**0.004**

Table S12. Individual ascending thoracic aortic diastolic dilation rates for OVX female mice. Absolute dilation rate (mm)/day, percentage dilation rate (%)/day and adjusted (adj) dilation rate(mm/mm)/day are shown.

Diastolic dilation rates					
**Mouse ID**	**Absolute rate (mm/day)**	**Diam at 30 days**	**Percentage rate (%/day)**	**Adj baseline diam**	**Adj rate (mm/mm/day)**
39030-2F	0.038	1.290	2.925	1.461	0.040
39053-1F	0.018	1.380	1.285	1.461	0.019
39060-2F	0.021	1.530	1.359	1.461	0.020
39060-5F	0.015	1.520	0.976	1.461	0.014
39085-3F	0.017	1.600	1.071	1.461	0.015
39115-1F	0.039	1.850	2.126	1.461	0.034
39125_1F	0.038	1.800	2.100	1.461	0.033
39149_1F	0.021	1.280	1.625	1.461	0.023
39169_1F	0.030	1.630	1.840	1.461	0.033
39161_1F	0.031	1.270	2.467	1.461	0.029
39187_1F	0.021	1.620	1.272	1.461	0.018
**Mean**	**0.026**	**1.525**	**1.732**		**0.0253**
**SD**	**0.009**	**0.193**	**0.591**		**0.008**

Table S13. Individual ascending thoracic aortic systolic dilation rates for placebo male mice. Absolute dilation rate (mm)/day, percentage dilation rate (%)/day and adjusted (adj) dilation rate(mm/mm)/day are shown.

Systolic dilation rates					
**Mouse ID**	**Absolute rate (mm/day)**	**Diam at 30 days**	**Percentage rate (%/day)**	**Adj baseline diam**	**Adj rate (mm/mm/day)**
39031-1M	0.020	1.530	1.307	1.619	0.023
39035-4M	0.055	1.460	3.744	1.619	0.060
39080-1M	0.056	1.710	3.294	1.619	0.053
39086-2M	0.013	1.580	0.827	1.619	0.014
39104-1M	0.025	1.690	1.499	1.619	0.023
39150-2M	0.017	1.470	1.156	1.619	0.022
39150-4M	0.030	1.360	2.181	1.619	0.038
39152-1M	0.060	2.240	2.679	1.619	0.040
39160-2M	0.052	1.710	3.021	1.619	0.049
39182_3M	0.013	1.620	0.794	1.619	0.013
39192-1M	0.044	1.690	2.604	1.619	0.042
**Mean**	**0.035**	**1.642**	**2.101**		**0.034**
**SD**	**0.018**	**0.220**	**0.990**		**0.015**

Table S14. Individual ascending thoracic aortic systolic dilation rates for 17β-Estradiol male mice. Absolute dilation rate (mm)/day, percentage dilation rate (%)/day and adjusted (adj) dilation rate(mm/mm)/day are shown.

Systolic dilation rates					
**Mouse ID**	**Absolute rate (mm/day)**	**Diam at 30 days**	**Percentage rate (%/day)**	**Adj baseline diam**	**Adj rate (mm/mm/day)**
39034-2M	0.009	1.470	0.617	1.619	0.014
39041-1M	0.009	1.560	0.573	1.619	0.011
39047-3M	0.013	1.330	0.952	1.619	0.022
39058-1M	0.010	1.600	0.604	1.619	0.010
39084-3M	0.008	1.450	0.570	1.619	0.014
39102-4M	0.013	1.780	0.712	1.619	0.008
39106-1M	0.043	1.770	2.437	1.619	0.038
39126-1M	0.006	1.710	0.324	1.619	0.003
39138-1M	0.018	1.750	1.017	1.619	0.014
39148_2M	0.013	1.750	0.739	1.619	0.009
39162-2M	0.006	1.620	0.362	1.619	0.006
39192A_2M	0.006	1.630	0.372	1.619	0.006
**Mean**	**0.013**	**1.618**	**0.773**		**0.013**
**SD**	**0.010**	**0.138**	**0.542**		**0.009**

Table S15. Individual ascending thoracic aortic systolic dilation rates for sham female mice. Absolute dilation rate (mm)/day, percentage dilation rate (%)/day and adjusted (adj) dilation rate(mm/mm)/day are shown.

Systolic dilation rates					
**Mouse ID**	**Absolute rate (mm/day)**	**Diam at 30 days**	**Percentage rate (%/day)**	**Adj baseline diam**	**Adj rate (mm/mm/day)**
39042-3F	0.004	1.420	0.268	1.619	0.010
39101-2F	0.015	1.560	0.974	1.619	0.026
39083-3F	0.009	1.290	0.698	1.619	0.011
39117-1F	0.014	1.590	0.872	1.619	0.015
39147-1F	0.006	1.480	0.374	1.619	0.010
39161-2F	0.005	1.510	0.353	1.619	0.009
39171_3F	0.014	1.480	0.977	1.619	0.019
**Mean**	**0.010**	**1.476**	**0.645**		**0.014**
**SD**	**0.005**	**0.092**	**0.286**		**0.006**

Table S16. Individual ascending thoracic aortic systolic dilation rates for OVX female mice. Absolute dilation rate (mm)/day, percentage dilation rate (%)/day and adjusted (adj) dilation rate(mm/mm)/day are shown.

Systolic dilation rates					
**Mouse ID**	**Absolute rate (mm/day)**	**Diam at 30 days**	**Percentage rate (%/day)**	**Adj baseline diam**	**Adj rate (mm/mm/day)**
39030-2F	0.034	1.560	2.175	1.619	0.036
39053-1F	0.014	1.560	0.902	1.619	0.026
39060-2F	0.016	1.720	0.942	1.619	0.024
39060-5F	0.016	1.610	0.994	1.619	0.020
39085-3F	0.014	1.710	0.830	1.619	0.021
39115-1F	0.031	2.000	1.567	1.619	0.019
39125_1F	0.036	1.970	1.831	1.619	0.025
39149_1F	0.011	1.460	0.776	1.619	0.027
39169_1F	0.020	1.800	1.111	1.619	0.025
39161_1F	0.022	1.450	1.517	1.619	0.016
39187_1F	0.016	1.750	0.895	1.619	0.011
**Mean**	**0.021**	**1.690**	**1.231**		**0.023**
**SD**	**0.008**	**0.176**	**0.446**		**0.006**
